# Vesicle shrinkage in hydrous phonolitic melt during cooling

**DOI:** 10.1007/s00410-020-1658-3

**Published:** 2020-02-12

**Authors:** A. Allabar, K. J. Dobson, C. C. Bauer, M. Nowak

**Affiliations:** 1 Department of Geosciences, University of Tübingen, Wilhelmstraße 56, 72074 Tübingen, Germany; 2 Department of Earth Sciences, Durham University, Durham, DH1 3LE UK; 3 Civil & Environmental Engineering, University of Strathclyde, Glasgow, G1 1XJ UK; 4 Department of Geosciences, University of Tübingen, Hölderlinstr. 12, 72074 Tübingen, Germany

**Keywords:** Decompression experiments, Vesiculation, Vesicle shrinkage, Quench effect, H_2_O resorption, Fictive temperature

## Abstract

The ascent of hydrous magma prior to volcanic eruptions is largely driven by the formation of H_2_O vesicles and their subsequent growth upon further decompression. Porosity controls buoyancy as well as vesicle coalescence and percolation, and is important when identifying the differences between equilibrium or disequilibrium degassing from textural analysis of eruptive products. Decompression experiments are routinely used to simulate magma ascent. Samples exposed to high temperature (*T*) and pressure (*P*) are decompressed and rapidly cooled to ambient *T* for analysis. During cooling, fluid vesicles may shrink due to decrease of the molar volume of H_2_O and by resorption of H_2_O back into the melt driven by solubility increase with decreasing *T* at *P* < 300 MPa. Here, we quantify the extent to which vesicles shrink during cooling, using a series of decompression experiments with hydrous phonolitic melt (5.3–3.3 wt% H_2_O, *T* between 1323 and 1373 K, decompressed from 200 to 110–20 MPa). Most samples degassed at near-equilibrium conditions during decompression. However, the porosities of quenched samples are significantly lower than expected equilibrium porosities prior to cooling. At a cooling rate of 44 K·s^−1^, the fictive temperature *T*_f_, where vesicle shrinkage stops, is up to 200 K above the glass transition temperature (*T*_g_), Furthermore, decreasing cooling rate enhances vesicles shrinkage. We assess the implications of these findings on previous experimental degassing studies using phonolitic melt, and highlight the importance of correctly interpreting experimental porosity data, before any comparison to natural volcanic ejecta can be attempted.

**Electronic supplementary material:**

The online version of this article (10.1007/s00410-020-1658-3) contains supplementary material, which is available to authorized users.

## Introduction

Volcanic eruptions are driven by magma density decrease caused by the exsolution of volatiles, mainly H_2_O (e.g., Gonnermann and Manga 2007). H_2_O supersaturation of the melt can be induced by a pressure (*P*) decrease and causes formation of vesicles, which then grow by both pressure related equation of state (EOS) expansion and continuous diffusion of H_2_O from the melt into the fluid phase (e.g., Sparks 1978). The porosity of a magma is a key parameter influencing the buoyancy and thus driving the acceleration of magma during ascent.

In experimental studies, the porosity of decompressed silicate melts subsequently quenched to glass has been used to investigate vesicle growth and coalescence as well as the evolution of permeability or percolation (Giachetti et al. 2019; Lindoo et al. 2016). Porosity has also been used to distinguish between equilibrium or disequilibrium degassing by comparing the glass porosity (*Φ*_glass_) or the residual H_2_O concentration in the glass (*c*_H2Oglass_) with those at experimental equilibrium conditions (e.g., Gardner 2012; Larsen and Gardner 2004; Mangan and Sisson 2000; Iacono-Marziano et al. 2007).

However, vesicles may shrink during cooling through a decrease in molar volume of H_2_O fluid (*V*_mH2O_) within the vesicles (EOS shrinkage; Marxer et al. 2015), and because of the increasing isobaric solubility of H_2_O in silicate melt with decreasing *T* at *P* < 300 MPa (Holtz et al. 1995; Schmidt and Behrens 2008), leading to resorption of H_2_O from the fluid vesicles back into the melt (McIntosh et al. 2014; Ryan et al. 2015). Together, these processes lead to a reduction in porosity, and increase the H_2_O concentration (*c*_H2O_) of the melt during cooling before the supercooled melt is quenched to a glass. Measured *Φ*_glass_ and* c*_H2Oglass_ therefore may not represent the molten state of the sample prior to cooling, especially when cooling rates are low. Slow cooling (~ 10 K·min^−1^) in sintering experiments using rhyolitic glass powder (*T* of 1023–823 K, *P* of 22 MPa and H_2_O concentrations up to ~ 2.2 wt%) in the presence of fluid leads to resorption of H_2_O vesicles resulting in fully dense obsidian (Gardner et al. 2019).

Cooling rates in decompression experiments are usually much faster (up to 150 K s^−1^), but *T* and *c*_H2O_ are significantly higher and melt viscosities are lower compared to the sintering experiments of Gardner et al. (2019). Consequently, vesicle shrinkage is still expected during cooling of vesiculated melts (McIntosh et al. 2014; Marxer et al. 2015; Allabar and Nowak 2018). McIntosh et al. (2014) have found that during fast cooling of experimentally decompressed phonolitic melt to ambient *T* within 3–10 s significant resorption occurs, demonstrating that the state prior to quench cannot be frozen in. Therefore, we further investigate this effect and quantify vesicle shrinkage and H_2_O resorption during cooling on an existing data set of vesiculated phonolitic melt with white pumice composition of the AD79 Vesuvius eruption (VAD79; Iacono-Marziano et al. 2007) quenched to glass. By applying different cooling rates (*q*) on these vesiculated phonolitic melts by additional experiments, we determine the influence of *q* on the extent of vesicle shrinkage.

Calculations were performed to quantify the effect of shrinkage during cooling, driven by the EOS of the H_2_O fluid and resorption of H_2_O back into the melt. To determine the fictive temperature (*T*_f_) where vesicle shrinkage stops, *Φ*_glass_ was used as well as the liquid water to vesicle volume ratio at ambient conditions derived from X-ray computed tomography (XCT) data. Finally, the results of this study are compared to previous decompression experiments with hydrous phonolitic melt and discussed with respect to the possible effect of vesicle shrinkage. Variables used in this publication are listed in Table 1.

**Table 1:** Symbol definitions

Symbol	Definition	Unit
*B*_s_	Vesicle shrinkage factor	
*c*_H2O_	H_2_O concentration	wt%
*c*_H2Oequ_	Equilibrium H_2_O concentration	wt%
*c*_H2Oglass_	H_2_O concentration in glass after quench	wt%
*c*_H2Oini_	Initial H_2_O concentration in the melt prior to decompression	wt%
*c*_H2OIR_	H_2_O concentration in the glass measured with FTIR close to vesicles	wt%
*c*_H2Ores_	Maximum possible resorbed *c*_H2O_ assuming resorption to *T*_g_	wt%
*c*_H2Ores_Tf_	Residual *c*_H2O_ at *T*_f_	wt%
*D*_H2O_	Diffusivity of H_2_O in silicate melt	mm^2^·s^−1^
d*P*/d*t*	Decompression rate	MPa·s^−1^
*l*	Characteristic diffusion length	mm
*P*	Pressure	MPa
*P*_final_	Final pressure where samples were quenched	MPa
*q*	Quench rate; NQ = 16 K s^−1^; MQ = 44 K s^−1^	K·s^−1^
*r*	Radius	m
*T*	Temperature	K
*T*_d_	Run temperature of decompression experiment	K
*T*_f_	Fictive temperature where vesicle shrinkage effectively stops	K
*T*_g_	Glass transition temperature	K
*T*_g_eq_	Glass transition temperature for melt with equilibrium *c*_H2O_	K
*T*_g_res_	Glass transition *T* for melt with maximum resorbed *c*_H2O_	K
*V*_H2Ol_	Volume of liquid H_2_O in vesicles at room *T*	µm^3^
*V*_mH2O_	Molar volume of H_2_O	cm^3^·mol^−1^
*VND*	Vesicle number density normalized to vesicle free sample volume	mm^−3^
*V*_ves_	Vesicle volume in the glass	µm^3^
Δ*P*_*PS*_	Difference between saturation *P* and *P* of phase separation	MPa
*η*	Melt viscosity	Pa·s
*η*_*_Tf*_	Viscosity at *T*_f_, where vesicle shrinkage stops	Pa·s
*ρ*_melt_	Melt density	g·cm^−3^
*σ*	Surface tension	N·m^−1^
*τ*_d_	Decompression timescale	s
*τ*_diff_	Diffusion timescale	s
*Φ*_EOS_	Calculated porosity when shrinkage works until *T*_g_eq_	%
*Φ*_equ_	Equilibrium porosity	%
*Φ*_glass_	Glass porosity	%
*Φ*_RES_	Calculated porosity when shrinkage works until *T*_g_res_	%

## Experimental and analytical methods

### Decompression experiments

We augment a series of decompression experiments from Allabar and Nowak (2018) and Allabar et al. (2020) (Table 2) to quantify vesicle shrinkage during cooling of vesiculated VAD79 phonolitic melts. The experiments of these studies were conducted in an internally heated argon pressure vessel (IHPV) at decompression temperatures (*T*_d_) of 1323–1373 K and initial dissolved H_2_O contents (*c*_H2Oini_) of 5.3, 4.3, and 3.3 wt%. Decompression rates were 0.064–1.7 MPa·s^−1^, starting from initial *P* of 200 MPa to final *P* (*P*_final_) ranging between 110–20 MPa. At *P*_final_, samples were cooled with a medium quench rate (MQ, although reported as RQ in Allabar and Nowak 2018). Samples were quenched by melting a platinum wire, at which the sample capsules were fixed during the experiments, leading to a capsule drop into the cold zone of the samples holder (Berndt et al. 2002) that was equipped with a brass rod at the bottom to reduce cooling rate (see “quantification of cooling rate”). This procedure was necessary to obtain intact samples for analysis.

**Table 2:** Summary of experimental conditions, results, and calculations

Sample	*c*_H2Oini_ [wt%]	*T*_d_ [K]	*P*_*final*_ [MPa]	d*P*/d*t* [MPa·s^−1^]	*q* [K·s^−1^]	log*VND* [mm^−3^]	*Φ*_glass_ [%]	*c*_H2OIR_ [wt%]	*c*_H2Oequ_ [wt%]	*τ*_diff_/*τ*_d_	*Φ*_equ_ [%]	*Φ*_EOS_ [%]	*Φ*_RES_ [%]	*T*_f_ [K]	*c*_H2Ores_Tf_ [wt%]	References
CD66	5.29 ± 0.11	1323	80	0.17	16 isob	5.22	0.5 ± 0.2	5.00 ± 0.11	3.12	5.7·10^–3^	26.9	8.7	0.0	683	5.23	this study
CD49	5.28 ± 0.09	1323	80	0.17	16 non isob	4.70	3.1 ± 0.3	n.d	3.12	8.8·10^–3^	26.9	8.7	0.0	767	4.74	this study
CD41	5.29 ± 0.09	1323	90	0.17	(44)*	5.22	1.1 ± 0.2	5.23 ± 0.05	3.34	6.8·10^–3^	22.7	–	–	753	5.11	this study
CD37	5.32 ± 0.09	1323	80	0.17	(16 non isob.)*	5.17	1.9 ± 0.2	5.08 ± 0.06	3.12	4.9·10^–3^	27.2	–	–	725	4.97	this study
CD74 (XCT)	5.33 ± 0.11	1323	70	0.17	44	n.d.	n.d.	n.d.	2.88	n.d.	32.3	12.1	1.9	810^ǂ^	4.22	this study
** ~ 5.3 wt%**																
CD40	5.17 ± 0.09	1323	100	0.17	44	5.15	0.1 ± 0.1	n.d.	3.56	1.9·10^–2^	17.9	5.8	0.0	784	5.21	AN18
CD42	5.20 ± 0.09	1323	90	0.17	44	4.98	1.1 ± 0.1	5.22 ± 0.06	3.34	9.8·10^–3^	21.9	7.3	0.0	768	5.03	AA20a
CD63	5.30 ± 0.10	1323	80	0.17	44	5.24	7.0 ± 0.7	5.10 ± 0.03	3.12	2.9·10^–3^	27.1	9.4	0.1	870	4.26	AN18
CD39	4.97 ± 0.13	1323	80	0.17	44	5.17	4.5 ± 0.5	5.00 ± 0.05	3.12	3.8·10^–3^	24.0	8.1	0.0	859	4.31	AN18
CD91	5.33 ± 0.10	1323	80	0.17	44	5.41	9.0 ± 1.1	n.d.	3.12	2.0·10^–3^	27.3	9.5	0.2	910	4.11	AA20a
CD83	5.46 ± 0.07	1323	80	0.17	44	5.78	3.2 ± 1.0	n.d.	3.12	1.6·10^–3^	28.5	10.0	0.6	745	4.86	AA20a
CD50	5.30 ± 0.09	1323	70	0.17	44	4.83	13.1 ± 1.4	5.18 ± 0.04	2.88	3.4·10^–3^	32.1	11.9	1.8	916	3.80	AN18
CD73	5.46 ± 0.17	1323	60	0.17	44	5.41	18.5 ± 2.2	n.d.	2.63	8.9·10^–4^	39.3	17.4	4.9	885	3.60	AA20a
CD92	5.10 ± 0.09	1323	80	0.064	44	5.35	4.6 ± 0.6	n.d.	3.12	1.1·10^–3^	25.2	8.6	0.0	839	4.39	AN18
CD78	5.12 ± 0.05	1323	82	1.7	44	5.32	3.3 ± 0.6	5.14 ± 0.08	3.16	3.5·10^–2^	24.5	8.2	0.0	810	4.59	AN18
CD28	5.29 ± 0.06	1323	95	1.7	44	5.05	0.5 ± 0.1	5.25 ± 0.04	3.45	1.3·10^–1^	20.8	6.8	0.0	755	5.24	AA20a
CD59	5.21 ± 0.05	1373	110	1.7	44	5.48	0.1 ± 0.1	n.d.	3.61	2.2·10^–1^	17.1	5.6	0.0	835	5.19	AA20a
CD55	5.22 ± 0.05	1373	100	1.7	44	4.60	0.5 ± 0.1	5.18 ± 0.02	3.40	3.9·10^–1^	20.5	6.7	0.0	791	5.17	AA20a
CD57	5.32 ± 0.07	1373	100	1.7	44	4.78	0.1 ± 0.1	5.32 ± 0.02	3.40	3.5·10^–1^	21.4	7.0	0.0	757	5.36	AA20a
CD51	5.27 ± 0.11	1373	75	1.7	44	4.98	4.2 ± 0.9	5.36 ± 0.07	2.85	5.1·10^–2^	31.5	11.5	0.7	776	4.54	AN18
CD52	5.21 ± 0.07	1373	90	0.17	44	4.96	1.6 ± 0.4	5.32 ± 0.02	3.19	1.0·10^–2^	24.2	8.0	0.0	784	4.94	AA20a
CD53	5.17 ± 0.06	1373	80	0.17	44	4.95	1.5 ± 0.2	5.21 ± 0.03	2.96	7.6·10^–3^	28.2	9.6	0.0	733	4.92	AN18
** ~ 4.3 wt%**																
CD87	4.31 ± 0.10	1323	60	0.17	44	5.51	5.4 ± 1.3	4.53 ± 0.17	2.63	8.0·10^–3^	27.8	11.1	0.0	852	3.72	AA20a
CD85	4.30 ± 0.13	1323	40	0.17	44	5.45	24.4 ± 2.2	3.91 ± 0.12	2.07	1.8·10^–3^	43.7	26.0	11.4	945	2.71	AA20a
** ~ 3.3 wt%**																
CD94	3.43 ± 0.05	1323	40	0.17	44	5.96	0.3 ± 0.1	n.d.	2.07	1.0·10^–2^	32.2	17.6	1.6	748	3.36	AA20a
CD95	3.36 ± 0.12	1323	20	0.17	44	6.37	15.4 ± 2.1	n.d.	1.37	1.1·10^–3^	58.5	45.4	35.2	646	2.65	AA20a

All decompression experiments were hydrated at 200 MPa prior to decompression. Symbol definitions are given in Table 1

^*^Quench rate of these samples was unknown after the experiment, but a possible quench rate was derived from *Φ*_glass_ (for details see text). Therefore, *Φ*_EOS_ and *Φ*_RES_ were not calculated for these samples; isob. = isobaric quench; non isob. = non isobaric quench with a *P* drop

^ǂ^*T*_f_ determined by XCT method

References: AN18: Allabar and Nowak (2018); AA20a: Allabar et al. (2020)

The experiments of this study were performed on the same starting material and at identical run temperature as in Allabar and Nowak (2018) and Allabar et al. (2020), with *c*_H2Oini_ of 5.3 wt% and a decompression rate of 0.17 MPa s^−1^. However, different quench protocols were applied, to investigate the influence on the *Φ*_glass_ of the finally analyzed glassy samples. Synthetic VAD79 glass cylinders with 5 mm diameter and 6.5 mm length were inserted together with 5.3 wt% H_2_O into Au80Pd20 tubes (13 mm length, 5 mm inner diameter) that were welded shut with a lid at the bottom. After sample filling, the upper ends of the capsules were crimped to a three sided star and welded. Possible leakage was checked by storing the capsules in a compartment dryer at 383 K, pressurizing to 100 MPa at ambient *T*, and again storing at 383 K. The capsules were re-weighed after each step. Constant capsule weight ruled out leakage and qualified the capsules for the experiments.

The samples were hydrated in the IHPV at slightly H_2_O undersaturated conditions at 200 MPa and superliquidus *T* of 1523 K for at least 94 h to obtain a homogeneous hydrous melt. After hydration, *T* was decreased to the decompression temperature *T*_d_ of 1323 K, still above the liquidus (Iacono-Marziano et al. 2007; Marxer et al. 2015; Allabar et al. (2020). The thermal gradient, measured by two thermocouples close to the samples over a distance of ~ 12 mm, is < 20 K. The *T* gradient within the samples is assumed to be < 10 K because the sample length of 6.5 mm is lower than the distance between the two thermocouples. The samples were equilibrated at *T*_d_ for at least 0.5 h before decompression was initiated. For the first pair of experiments, samples were decompressed from 200 MPa to *P*_final_ of 80 MPa and quenched by switching off the furnace, while the samples remained in the hot zone of the sample holder (normal quench = NQ). The sample CD49 underwent a non-isobaric NQ, with a monitored *P* drop of ~ 5–7 MPa during cooling of the argon pressure medium (NQ non-isobaric), while sample CD66 underwent an isobaric NQ (± 0.1 MPa), with *P* being kept constant by pumping additional argon into the vessel. Sample CD74 (a replicate of CD50; Allabar et al. (2020), was decompressed to 70 MPa and quenched with MQ. An additional pair of experiments, samples CD37 and CD41, were decompressed to 80 and 90 MPa, respectively, then cooled with an unknown rate that must have been either non-isobaric NQ or MQ, because of a missing *T* decrease that would have indicated the capsule drop from the hot zone into the cool part of the sample holder. After re-weighing to exclude capsule leakage, the quenched samples, except of CD74, were cut along their cylinder axis. One half of each sample was prepared for SEM image analysis and the other halves were prepared to obtain double-sided polished thin sections with 89–210 µm thickness for Fourier Transform Infrared (FTIR) and transmitted light microscopy analysis. Thin section thickness was measured with a Mitutoyo digital micrometer (± 3 µm). Sample CD74 was unwrapped from the capsule material and scanned using XCT.

### Quantification of cooling rate

For the IHPV with rapid quench setup, a cooling rate (*q*) of ~ 150 K·s^−1^ (RQ) was determined by Berndt et al. (2002) for dropping the capsule from the hot zone of the furnace into the cold part of the samples holder. The temperature at the bottom of the sample holder is 293–298 K at experimental *T* of up to 1523 K and 200 MPa (Berndt et al. 2002). However, for experiments from Allabar and Nowak (2018) and Allabar et al. (2020) the aim was to reduce tension crack formation in the samples during quench. Otherwise, samples will likely disintergrate and pieces may be lost during preparation. Thus, the experimental setup of the IHPV sample holder was modified by inserting a 35 mm brass cylinder at the bottom of the sample holder. This setup enables a medium quench rate (MQ) because the capsule stays slightly closer to the hot zone of the furnace during cooling, i.e., at *T* > 298 K. In order to quantify the quench rate of this setup, reference experiments where performed on two glass cylinders of the same geometry as for decompression experiment samples (5 mm diameter, 6.5 mm length) using haplogranite composition (AOQ = Ab_38_Or_34_Qz_28_, Holtz et al. 1995; Nowak and Behrens 1995). The AOQ melts were hydrated with ~ 5.3 wt% H_2_O in Au80Pd20 capsules at 200 MPa and 1523 K for 96 h. After hydration, one sample experienced the isobaric MQ protocol, while the other underwent an isobaric NQ. Doubly polished thin sections were prepared from the quenched glasses and the molecular H_2_O and OH group absorption band intensities determined with FTIR (A_5230_ and A_4520_, respectively). 20 and 23 measurements were performed on the NQ and RQ sample, respectively, with a spectral resolution of 4 cm^−1^ and 50 scans per measurement. A linear baseline subtraction (Behrens et al. 1996) and normalization to 1 mm sample thickness was applied. Cooling rates were then determined using the hydrous species geospeedometer (Zhang et al. 2000). For the MQ protocol, mean *A*_5230_ = 0.793 ± 0.08 and *A*_4520_ = 0.343 ± 0.003 yield a cooling rate of 44 ± 11 K·s^−1^. For the NQ protocol, mean *A*_5230_ = 0.769 ± 0.002 and *A*_4520_ = 0.330 ± 0.002 give a cooling rate of 16 ± 3 K·s^−1^. This NQ cooling rate is consistent with the logged *T* close to the sample during cooling, which showed nearly linear cooling from *T*_d_ down to ~ 573 K. The glass transition temperature (*T*_g_) of this AOQ glass (at 5.3 wt% and a cooling rate of 16 K·s^−1^) is at 660 K (Dingwell and Webb 1990; Giordano et al. 2008). Thus, linear cooling down to *T*_g_ can be assumed. The near sample *T* logs during NQ at *P* between 200 and 50 MPa show that the cooling rate is nearly independent of *P* in this range.

### Determination of vesicle number density and glass porosity

Vesicle number density (*VND*) and *Φ*_glass_ were determined merely on the vesiculated central sample volume because this volume is of interest to study vesiculation driven by homogeneous phase separation. Heterogeneously nucleated fringe vesicles at the capsule wall and the drainage zone that is formed by diffusional loss of H_2_O into fringe vesicles are experimental artifacts (e.g., Mangan and Sisson 2000; Iacono-Marziano et al. 2007; Preuss et al. 2016; Allabar and Nowak 2018; Allabar et al. 2020) and were therefore omitted for analysis.

Sample CD66 was analyzed with transmitted light microscopy on the thin section of the sample. Vesicles were counted during focusing through the sample and *Φ*_glass_ was determined using measured vesicles sizes. The *VND*s in the other samples of this study were determined by analysis of the backscattered electron (BSE) images, because vesicles are large enough for a sufficiently high intersection probability. The BSE images were analyzed using ImageJ and the stereological 2–3D conversion using CSDCorrections (Higgins, 2000). The *VND* was normalized to vesicle free glass volume. An error in *VND* of ± 0.13 log units was estimated with an error propagation considering all steps that are prone to errors (Allabar et al. 2020). Errors of *Φ*_glass_ were provided by CSDCorrections in case of BSE image analysis. For transmitted light microscopy analysis, an error in *Φ*_glass_ was estimated with an error propagation calculation, using the error in sample thickness of ± 3 µm and assuming an error of 10% for vesicle size measurement and 5% for the vesicle count. A more detailed description of *VND* and *Φ*_glass_ analysis is given in Marxer et al. (2015) and Allabar et al. (2020).

### FTIR micro-spectroscopy

**Fig. 1:**
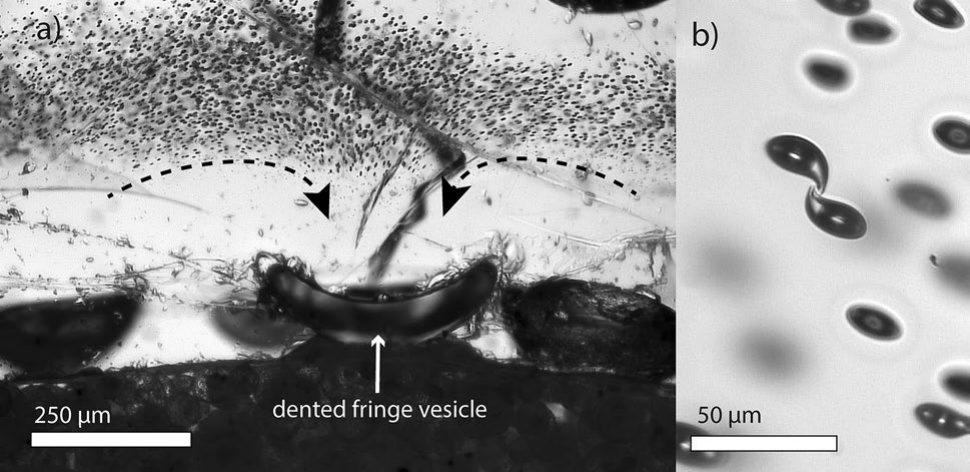
**a** Transmitted light microscopy image of sample bottom of CD50 (Allabar and Nowak 2018). The fringe vesicles are dented and flow textures in the zone with small vesicles are visible. Large vesicles are surrounded by a vesicle free drainage zone. **b** Transmitted light microscopy image of vesicles within the central part of CD49. Vesicles positioned close to each other are often deformed and form a neck towards each other. Detailed capsule images are shown in the online resources of Allabar et al. (2020)

Near-infrared measurements in transmission mode were carried out with a Hyperion 3000 IR-microscope connected to a Bruker Vertex v80 FTIR, using a CaF_2_ beam splitter and an InSb single element detector together with a 15 × Cassegrain objective. Samples of Allabar et al. (2020) and samples of this study were measured to determine the residual *c*_H2O_ in the glass between vesicles (*c*_H2OIR_). Measurements were located between vesicles in the central sample volume or as close as possible to the vesicles at the margin of the central vesiculated volume (Fig. 1). The knife-edge aperture was adjusted for each measurement individually between 10 × 10 to 30 × 30 µm to ensure that the beam path was free of vesicles. This was checked by focusing through the entire sample volume in *z* direction at the measurement location. 50 scans were recorded per spectrum with a spectral resolution of 4 cm^−1^ using air as reference. At least five spots per sample were measured. For the determination of *c*_H2OIR_ from molecular H_2_O (A_5210_) and hydroxyl group absorbance (A_4470_) the *c*_H2O_-density relationship (*ρ*[g·cm^−3^] = 2.47–0.013·*c*_H2O_ [wt%]) and linear molar extinction coefficients (*ε*_H2O_ = 1.18 and *ε*_OH_ = 1.14 l·mol^−1^ cm^−1^) from Iacono-Marziano et al. (2007) were used.

To resolve *c*_H2O_ gradients with high spatial resolution between vesicles in the central vesiculated part of the sample CD73, mid-infrared measurements were performed using an attenuated total reflectance (ATR) objective (20x) mounted on the Hyperion3000 microscope in combination with a 64 × 64 focal plane array detector (FPA). The ATR germanium (Ge) crystal was brought in contact with the polished sample surface using a contact pressure of 3 (within a possible range of 1–5; ~ 4.4 N). At higher contact pressures the vesiculated glasses tend to break. A value of 3 is low enough to prevent cracking and is sufficient for reproducible peak heights of the fundamental OH stretching band at 3450 cm^−1^. An Au-mirror was used as reference, spectral resolution was decreased to 16 cm^−1^ to minimize the noise associated with atmospheric water vapor (Lowenstern and Pitcher 2013) and 256 scans were accumulated for each spectrum. With these settings, 128 × 128 spectra were collected in 4 frames that cover a sample area of 70 × 70 µm. For each spectrum the peak height of the fundamental OH stretching band at 3450 cm^−1^ was determined after linear baseline subtraction anchored at 3760 and 2430 cm^−1^. Calibration for absolute *c*_H2O_ for this method was not performed because the contact pressure, i.e., the energy of the light reaching the detector across the area of a frame, was not homogeneous. This is probably due to a tiny tilt of the Ge-ATR-crystal relative to the sample surface.

### Raman micro-spectroscopy

Raman micro-spectroscopy (RMS) mapping was performed on sample CD73 at the same location as the FTIR mapping to quantify *c*_H2Oglass_ gradients. A Renishaw InVia confocal Raman micro-spectrometer was used, equipped with a 532 nm (“green”) laser, a 1800 grooves·mm^−1^ grating, and a Peltier-cooled CCD-detector. Laser power was set to 10%, corresponding to ~ 2.5 mW on the sample, which is low enough to avoid oxidation or dehydration of the samples (Di Genova et al. 2017). A 50 × objective with a numerical aperture of 0.75 was used together with the high confocality setting to optimize for spatial resolution. The laser was focused at the sample surface, and it was checked whether the laser remained focused while moving across the mapped area. An area of 38 × 52 µm was mapped with one spectrum collected each µm (= 1976 spectra) from 100–4000 cm^−1^ with an acquisition time of 10 s. Laser intensity was checked before and after the mapping to confirm its stability. To obtain total H_2_O concentration from the Raman spectra, a calibration was performed with hydrated reference samples from Marxer et al. (2015) (REF02-06 and one unpublished sample) with known *c*_H2O_ (1.37–5.60 wt%) from FTIR spectrsocopy. Two calibrations were used (e.g., Schiavi et al 2018): (1) calibration of the high wavenumber 3450 cm^−1^ band (HW) and (2) calibration of the ratio of the HW band and the low wavenumber (LW, 200–1250 cm^−1^) alumino-silicate bands. A detailed description of calibration is given in the online resource “RMS and FTIR supplementary information”.

### XCT-measurements

The MQ sample (CD74) was scanned using a Zeiss Xradia 410 laboratory scanner system operating at 130 kV, a current of 76 µA, and the Xradia HE3 filter to reduce beam hardening effects. 2001 projections were collected with an exposure time of 10 s and the data were reconstructed using the Xradia proprietary algorithm to give a data volume with voxels of 2.06 µm edge length.

The image data were segmented and visualized using AVIZO© (ThermoFisher). After first defining a sample mask (manual refinement of a magic wand segmentation interpolated between every 100th slice to ensure capture of all edge contacting vesicles with thin films), the image data were segmented into glass and vesicle using the magic wand tool built into the Avizo segmentation workroom. Image noise and the smallest vesicle population were then removed by opening (kernel = 3) and closing (kernel = 3) and a 2D (perpendicular to sample axis) fill operation. Segmentation of the vesicle-gas and vesicle-liquid phases is challenging because of the small difference in greyscale value, and the low signal–noise ratio in the raw data. However, after applying a 3D non-local means filter (5, 0.2, 10. 3) to the vesicles, segmentation could be achieved manually with a single click per vesicle. For comparison, two vesicles were segmented by manual assignment to the liquid and gas phase, before the volume of the different phases were calculated.

## Results and discussion

In the samples of this study, we observed heterogeneously nucleated fringe vesicles attached to the capsule walls, a vesicle free drainage zone, and a finely vesiculated central volume formed by homogeneous phase separation as reported elsewhere (e.g., Iacono-Marziano et al. 2007; Allabar and Nowak 2018; Allabar et al. 2020). The log*VND* (in mm^−3^) in the central sample volume ranges between 4.70 and 5.22 (analytical error ± 0.13 log units; Allabar et al. 2020), which is consistent with the log*VND* observed in other samples with a *c*_H2Oini_ of ~ 5.3 wt% (Table 2). Sample capsules after decompression and quench are often deformed with concave capsule walls. Furthermore, the fringe vesicles in all samples including those of Allabar et al. (2020) are strongly deformed with melt flow textures in their vicinity sometimes marked by small vesicles (Fig. 1a). The homogeneously distributed small vesicles in the sample center are spherical, except of few vesicle pairs where the inter vesicle wall is dimpled or a neck is formed from one vesicle to the other (Fig. 1b). Castro et al. (2012) explain such textures with the onset of coalescence, while McIntosh et al. (2014) attribute this to vesicle shrinkage during cooling.

Marxer et al. (2015) have also reported collapsed capsules with concave deformed walls after the decompression experiments (Figs. 6 and 15b, therein) and attribute this to vesicle shrinkage during cooling. Cooling causes a *P* drop within the capsule relative to quench pressure that drives capsule collapse during cooling. In contrast to this observation, an experimental setup capable to record the complete volume increase during decompression-induced degassing should freeze melt porosity (*Φ*_melt_) at *P*_final_ during cooling. Consequently, capsules should show a convex shape. A concave deformation, however, as observed by Marxer et al. (2015) and in the experiments summarized in this study, can only be explained by a volume reduction of the capsule interior, i.e., shrinkage of H_2_O vesicles, during cooling. Thus, we suggest that the textures observed at ambient conditions record vesicle shrinkage during cooling. These textures include the collapse of large fringe vesicles and flow textures preserved in the finely vesiculated glass in their vicinity. Strongly deformed vesicles attached to the capsule wall would not be able to maintain a spherical shape if shrinkage were faster than the time required for the vesicle to adjust its geometry by reduction of surface energy. In the central sample volume, a low number of large vesicles (~ 200–300 µm diameter) that appear at low *P*_final_ (sample CD 73) preserve deformation (Allabar et al. 2020) while the small vesicles (~ 15 µm diameter) within the central sample volume are nearly spherical. The relaxation of deformed vesicles to a spherical shape depends on the radius (Toramaru, 1998):

1 $$t_{{{\text{relax}}}} = \frac{r \cdot \eta }{\sigma }$$

where *r* is radius in m, *η* is viscosity in Pa s and *σ* is surface tension in N m^−1^. Thus, *t*_relax_ is more than 10 times larger for a vesicle 250 µm in diameter than for a vesicle with 15 µm diameter. However, a quantitative calculation is not possible here, because the quenched vesicle sizes do not represent the changing radii in the cooling melt, and in addition, *η* undergoes a complex evolution because of simultaneous cooling and melt rehydration.

### Quantification of melt porosity prior to quench

The textural observations of the samples indicate vesicle shrinkage during cooling. Therefore, it is assumed that *Φ*_glass_ and *c*_H2OIR_ do not represent *Φ*_melt_ and *c*_H2O_ of the melt prior to cooling, respectively. Thus, *Φ*_melt_ and *c*_H2O_ at *P*_final_ and *T*_d_ are initially unknown. Nevertheless, the relative importance of H_2_O diffusion during decompression can be determined, which is quantified by the ratio of diffusion timescale *τ*_diff_ to the timescale of decompression *τ*_d_ (Hajimirza et al. 2019). If the diffusion timescale is shorter than the decompression timescale (*τ*_diff_/*τ*_d_ << 1), near-equilibrium degassing is facilitated and the melt porosity and *c*_H2O_ prior to quench can be calculated.

In the decompression experiments with hydrous phonolitic melt, the vesicles in the central melt volume form in a single event (Allabar and Nowak 2018; Allabar et al. 2020), so *τ*_d_ is the time between the *P* at which phase separation occurs and *P*_final_. The *τ*_diff_ is defined as

2 $$\tau_{{{\text{diff}}}} \equiv \frac{{l^{2} }}{{D_{{{\text{H}}_{{2}} {\text{O}}}} }}$$

where *D*_H2O_ is the total H_2_O diffusivity in mm^2^ s^−1^ as a function of *c*_H2O_ in wt% and *T* in K, calculated after Fanara et al. (2013):

3 $$\log D_{{{\text{H}}_{2} {\text{O}}}} = - 1.11 - 2.07\log c_{{{\text{H}}_{2} {\text{O}}}} - \frac{{\left( {4827 - 4620\log c_{{{\text{H}}_{2} {\text{O}}}} } \right)}}{T}$$

The characteristic diffusion length *l* in mm is defined by the inter-vesicle distance calculated as follows (Hajimirza et al. 2019):

4 $$l = \frac{{1 - \left( {\frac{\varPhi }{100}} \right)^{\frac{1}{3}} }}{{\left( {1 - \frac{\varPhi }{100}} \right)^{\frac{1}{3}} }}\left( {\frac{4\varPhi }{3}{\text{VND}}} \right)^{{ - \frac{1}{3}}}$$

For a conservative estimate of *l* we use *Φ*_glass_ for calculations. A conservative estimate of *D*_H2O_, and thus of *τ*_diff_, is realized using the equilibrium *c*_H2O_ (*c*_H2Oequ_) at the *P*_final_ of each experiment, which results in the slowest possible H_2_O diffusivity. The *c*_H2Oequ_ at 1373 K for VAD79 phonolitic melt (Iacono-Marziano et al. 2007; Marxer et al. 2015) is calculated by:

5 $$c_{{{\text{H}}_{2} {\text{O}}}}^{{1323 {\text{K}}}} \left[ {{\text{wt}}\% } \right] = 0.2321 \cdot P\left[ {{\text{MPa}}} \right]^{0.5928}$$

For the onset of vesiculation, we use 110, 70, and 50 MPa for samples with 5.3, 4.3, and 3.3 wt% *c*_H2Oini_, respectively, to calculate *τ*_d_. The given *P’*s are 10 MPa above the respective *P*_final_, at which vesicles are observed in the vitrified samples. It may be possible that vesiculation starts earlier at higher *P* and vesicles were completely resorbed in the high *P*_final_ experiments (see argumentation below). However, the usage the given *P*’s constitutes a conservative estimate for *τ*_d._ The obtained *τ*_diff_ /*τ*_d_ values are all << 1 (Table 2) suggesting near-equilibrium degassing during decompression prior to quench. The resulting equilibrium melt porosity at *P*_final_ (*Φ*_equ_) prior to quench can be calculated (Gardner et al. 1999, Eq. 5 therein):

6 $${\varPhi}_{{{\text{equ}}}} = \left[ {\frac{{\frac{{\rho_{{{\text{melt}}}} }}{{M_{{{\text{H}}_{2} {\text{O}}}} }} \cdot V_{mH2O} \cdot \left( {c_{{{\text{H}}_{{2}} {\text{Oini}}}} - c_{{{\text{H}}_{{2}} {\text{Oequ}}}} } \right)}}{{1 + \left( {\frac{{\rho_{{{\text{melt}}}} }}{{M_{{{\text{H}}_{2} {\text{O}}}} }} \cdot V_{mH2O} \ \cdot \left( {c_{{{\text{H}}_{{2}} {\text{Oini}}}} - c_{{{\text{H}}_{{2}} {\text{Oequ}}}} } \right)} \right)}}} \right]$$

where melt density *ρ*_melt_ (Ochs and Lange 1999) and molar volume of H_2_O fluid *V*_mH2O_ (Duan and Zhang 2006) are calculated for equilibrium conditions at *P*_final_ prior to quench (Table 2). Furthermore, near-equilibrium degassing is evidenced by similar *Φ*_glass_ observed in the central volumes of samples decompressed with different d*P*/d*t* to a similar *P*_final_. The samples with ~ 5.3 wt% *c*_H2Oini_ that are decompressed at *T*_d_ of 1323 K to 80–82 MPa reveal similar *Φ*_glass_ of 3.3–7.0% with no systematic dependence on d*P*/d*t* in the range between 0.064–1.7 MPa·s^−1^ (Allabar and Nowak 2018). If disequilibrium degassing occurred prior to quench, significant increase in porosity with decreasing d*P*/d*t* at constant *P*_final_ would be expected, which is not observed here.

Since near-equilibrium degassing must have prevailed in the experiments, melt porosity equals *Φ*_equ_ and *c*_H2O_ of the melt equals *c*_H2Oequ_ prior to quench. This knowledge enables us to quantify vesicle shrinkage during cooling.

### Vesicle shrinkage during cooling

**Fig. 2:**
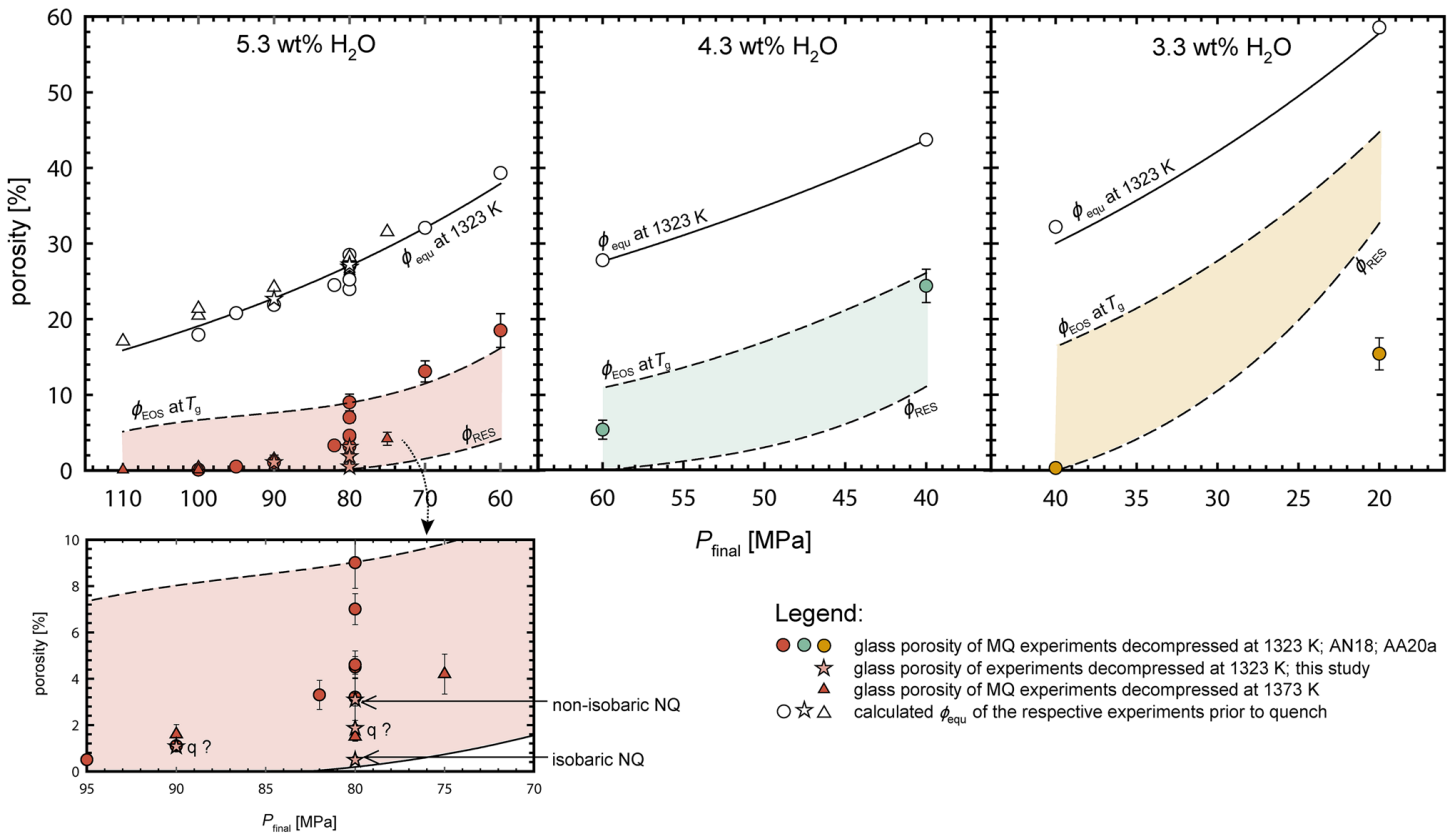
Observed glass porosities (*Φ*_glass_) vs. *P*_final_ for 5.3, 4.3 and 3.3 wt% *c*_H2Oini_. Equilibrium porosity (*Φ*_equ_, solid line) is calculated for 1323 K and 5.3, 4.3 and 3.3 wt% *c*_H2Oini_, respectively. The deviation of individual equilibrium porosity from the *Φ*_equ_ curve originates from slight variation in initial H_2_O concentration or a slightly increased decompression temperature of 1373 K (triangles). *Φ*_EOS_ represents the calculated porosity at *T*_g_ related to EOS-shrinkage. *Φ*_RES_ is the minimum possible porosity taking additionally into account that H_2_O is resorbed during cooling until *T*_g_res_ is reached, according to the H_2_O solubility model of Shea (2017) (Eq. 8). Both *Φ*_EOS_ and *Φ*_RES_ are calculated for a cooling rate of 44 K s^−1^. For details see text. The lower box is a zoomed excerpt of the 5.3 wt% *c*_H2Oini_ experiments for detailed visualization. References: AN18: Allabar and Nowak 2018; AA20a: Allabar et al. (2020)

For all samples, the *Φ*_glass_ observed at ambient conditions (0.1–24.4%) is significantly lower than the calculated *Φ*_equ_ prior to quench (17.1–58.5%, Table 2, Fig. 2). This is in line with *c*_H2OIR_ at ambient conditions (3.91–5.36 wt%) that are higher than *c*_H2Oequ_ prior to quench (1.37–3.61 wt%, Table 2). These discrepancies suggest that both significant vesicle shrinkage and H_2_O resorption occurred in the experimentally decompressed samples during cooling.

The extent of vesicle shrinkage increases with lower *q*, as demonstrated by sample CD66 (*c*_H2Oini_ = 5.22 wt%; *P*_final_ = 80 MPa; *q* = 16 K·s^−1^ isobaric), which has a lower *Φ*_glass_ of 0.5% compared to MQ experiments with identical *P*_final_ and similar *c*_H2Oini_ (*q* = 44 K·s^−1^; *Φ*_glass_ = 1.5–9.0%). With slow cooling, more time is available for diffusion to resorb H_2_O from the fluid back into the melt, and for the vesicle size to equilibrate to the decreasing internal vesicle *P* by viscous flow of the melt. The *Φ*_glass_ of 3.1% in CD 49 (*q* = 16 K·s^−1^ non-isobaric) is in the range of porosities of the MQ experiments. Vesicles of this sample shrunk less than expected for isobaric quench because the influence of decreasing *P* during cooling on *V*_mH2O_ and H_2_O solubility in the melt dampens the effect of *T* decrease. When *Φ*_glass_ of the experiments CD41 and CD37 with unknown *q* are compared to the trend of experiments with 44 K·s^−1^ (Fig. 2), it can be estimated that CD41 (*P*_final_ = 90 MPa; *Φ*_glass_ = 1.1%) experienced a *q* of 44 K·s^−1^ and CD37 (*P*_final_ = 80 MPa; *Φ*_glass_ = 1.9%) a non-isobaric *q* with 16 K·s^−1^.

### Quantification of vesicle shrinkage during cooling

Vesicle shrinkage can be calculated when the conditions prior to quench are known. Note that resorption shrinkage requires *c*_H2Oequ_ being adjusted in the melt prior to cooling (Ryan et al. 2015). Otherwise, when the cooling-induced increase of H_2_O solubility of the melt does not exceed H_2_O supersaturation during cooling, no driving force for resorption of H_2_O from the fluid phase back into the melt evolves. In our experiments both EOS- and H_2_O resorption-shrinkage occurred because cooling started from *Φ*_equ_ and *c*_H2Oequ_.

To demonstrate the contribution of each vesicle shrinkage mechanism to the overall shrinkage, we calculate in a first step solely EOS shrinkage down to *T*_g_ without any resorption (Marxer et al. 2015). For the melt with *c*_H2Oequ_ at the respective *P*_final_, *T*_g_ was calculated (= *T*_g_equ_) using the viscosity model of Giordano et al. (2008) and the cooling rate (44 K·s^−1^) dependent viscosity that defines the glass transition (log *η* = 9.66 Pa·s; Dingwell and Webb 1990) where vesicle shrinkage is assumed to stop. The *V*_mH2O_ at *P*_final_ and *T*_d_ or *T*_g_equ_ can then be calculated (EOS of H_2_O, Duan and Zhang (2006) for *T* > 673.15 K; WaterSteamPro™ (Orlov et al. 1999-2020 Version 6.5.0.64) for *T* < 673.15 K). The ratio of *V*_mH2O_(*P*_final_, *T*_run_) and *V*_mH2O_(*P*_final_, *T*_g_equ_) corresponds to the shrinkage factor *B*_s_ (Marxer et al. 2015). This factor is then used to calculate the glass porosity by Eq. 7 (Marxer et al. 2015, Eq. 4 therein) at *T*_g_ (*Φ*_EOS_, Fig. 2) when shrinkage started from *Φ*_equ_:

7 $${\varPhi }_{\mathrm{E}\mathrm{O}\mathrm{S}}=\frac{{\varPhi }_{\mathrm{e}\mathrm{q}\mathrm{u}}}{{\varPhi }_{\mathrm{e}\mathrm{q}\mathrm{u}}-{B}_{s}\cdot ({\varPhi }_{\mathrm{e}\mathrm{q}\mathrm{u}}-100)}\cdot 100$$

However, this calculated *Φ*_EOS_ at *T*_g_ likely underestimates the real value, because the vesicles will effectively stop shrinking at a temperature > *T*_g_ due to limited viscous flow with increasing melt viscosity.

**Fig. 3:**
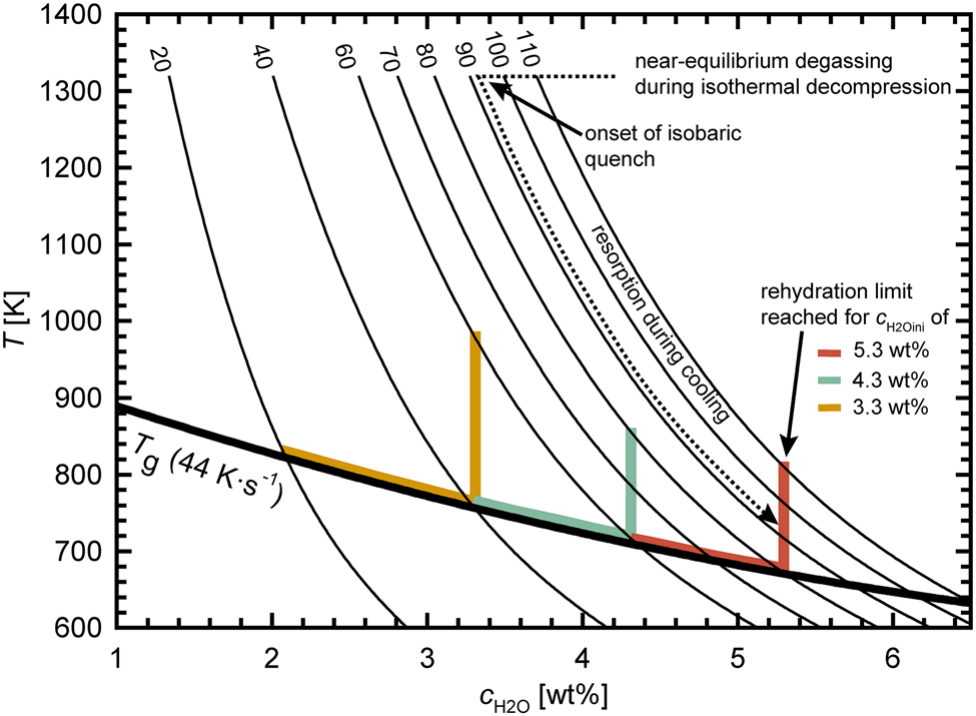
Rehydration quench scenarios using the method of Ryan et al. (2015) for *P*_final_ of the decompression experiments with *c*_H2Oini_ of 5.3–3.3 wt%, summarized in Table 2. Isobaric H_2_O solubility curves (thin black lines) for different *P* (in MPa) are calculated, using the H_2_O-solubility equation for phonolitic melt from Shea et al. (2017) (Eq. 8). The *T*_g_ curve (thick black line) as a function of *c*_H2O_ for *q* = 44 K·s^−1^ was calculated with the viscosity model of Giordano et al. (2008) accounting for quench rate dependence of *T*_g_ (Dingwell and Webb 1990). The colored lines indicate the rehydration limit for the experiments summarized in Table 2 defined by maximum possible resorbed *c*_H2O_. These lines follow the *T*_g_ curve as long as the maximum resorbed *c*_H2O_ is < *c*_H2Oini_. When the cooling-induced solubility increase reaches *c*_H2Oini_ (vertical colored lines), rehydration can be completed, and all vesicles will be fully resorbed if diffusion time is sufficient

In a second step, we additionally account for resorption shrinkage because sample porosity is further reduced by H_2_O resorption during cooling. Therefore, combined shrinkage by EOS and H_2_O resorption was calculated using the rehydration quench method of Ryan et al. (2015) (Fig. 3). Here, it is considered that *T*_g_ continuously decreases during cooling due to the H_2_O resorption of melt (i.e., viscosity reduction). Ultimately, the melt is quenched to a glass when the *T*_g_ curve and retrograde H_2_O solubility curve intersect (= *T*_g_res_; Fig. 3) defining the residual *c*_H2O_ in the glass after maximum possible resorption (*c*_H2Ores_). Here, the *T*-dependent solubility equation for phonolite from Shea et al. (2017) (which reproduces *c*_H2Oequ_ at 1323 K from Eq. 5 with < 3% relative deviation) was used to calculate temperature dependent solubility ${c}_{{H}_{2}{O}}$ curves during isobaric cooling (Fig. 3):

8 $$c_{{{\text{H}}_{2} {\text{O}} }} = \frac{{330P^{0.5} + 16P - 1.6P^{1.5} }}{T} + 0.001\;P^{1.5}$$

where *P* is in MPa and *T* in K. The *T*_g_ curve was calculated for a cooling rate of 44 K·s^−1^ (Giordano et al. 2008; Dingwell and Webb 1990). Each cooling path is defined by the solubility curve, assuming that resorption effectively occurs until *T*_g_res_. When the solubility reaches the *c*_H2Oini_ of the sample, *T*_g_res_ is equal to *T*_g_ of the melt with *c*_H2Oini_. In this case, the degassed H_2_O can be fully resorbed when the melt attains equilibrium H_2_O content during cooling.

The possibility of complete resorption has consequences for experiments where the pressure difference required for (heterogeneous or homogeneous) phase separation (Δ*P*_PS_) is intended to be determined. Δ*P*_PS_ is usually determined by finding the *P* at which vesicles are observable for the first time after decompression. If quench rates are used that allow vesicle shrinkage, vesicles may be fully resorbed until a certain pressure is reached and as a result Δ*P*_PS_ can be significantly overestimated by post mortem analysis of the vesiculated glasses. However, in this case, independent Δ*P*_PS_ estimates might be useful, e.g., using the widths of the drainage zone in quenched samples to calculate the time needed to develop the diffusion width of the zone were no vesicles were formed due to insufficient supersaturation (Allabar and Nowak 2018) or by performing in-situ decompression experiments (Masotta and Keppler 2017). In turn, for all samples quenched at *P* for which the solubility curves in Fig. 3 cross the *T*_g_ curve instead of ending at the rehydration limit, it can be ensured that vesicles will be observable in these samples. Shrinkage and resorption could also decrease the *VND* observed in vitrified samples when small vesicles are resorbed while large vesicles are not resorbed completely. However, in the experiments studied here, vesicles are similarly sized and we do not observe an increase of *VND* with decreasing *P*_final_, suggesting that either all vesicles are resorbed to sizes below optical resolution or all vesicles remain at sizes above optical resolution during cooling.

With *T*_g_res_ and the respective *c*_H2Ores_ the maximum extent of shrinkage and thus the minimum possible glass porosity (*Φ*_RES_) was calculated (Fig. 2). *Φ*_EOS_ and *Φ*_RES_ define an area (Fig. 2), in which the glass porosity of the quenched experimental products are likely to be found, when they experienced equilibrium degassing prior to quench. At low *P*_final_, one reason why this area becomes narrower is that the slopes of isobaric solubility curves are higher (Fig. 3), which results in less resorption.

### Comparison of calculated with observed porosities

Within error, the *Φ*_glass_ of the experimental samples with *c*_H2O ini_ of ~ 5.3 and ~ 4.3 wt% plot between the calculated *Φ*_EOS_ and *Φ*_RES_ (Fig. 2). At high *P*_final_, the glass porosities follow the resorption trend and then approximate the EOS trend at low *P*_final_ and higher porosities. This can be attributed to the higher residual *c*_H2O_ in the melt at high *P*_final_ resulting in a faster H_2_O diffusivity (Eq. 3) e.g., by half an order of magnitude at 100 MPa as compared to 60 MPa, assuming equilibrium H_2_O content. Thus, resorption is expected to be more efficient at higher *P*_final_. The variation of *Φ*_glass_ of the experiments quenched at a *P*_final_ of 80 MPa can be explained by slight variations in *c*_H2Oini_, as it can also be seen in the variation of *Φ*_equ_ for the individual experimental samples as compared to the calculated *Φ*_equ_ as function of *P*_final_ for a sample with *c*_H2Oini_ of 5.3 wt% (Fig. 2).

The *Φ*_glass_ of sample CD94 with *c*_H2Oini_ of 3.3 wt% and *P*_final_ of 40 MPa is close to the calculated *Φ*_RES_ trend, which can be explained by the high log*VND* of 5.96. This value is almost one order of magnitude higher than the log*VND* of ~ 5 in the 5.3 wt% *c*_H2Oini_ experiments (Table 2). Despite the slower H_2_O diffusivity, the inter-vesicle distance (< 10 µm) in the 3.3 wt% experiments is roughly half of that of the 5.3 wt% experiments, which in turn improves efficiency of H_2_O resorption. This results in similarly low τ_diff_/τ_d_ for experiments with *c*_H2Oini_ of 3.3 wt% as compared to experiments with higher *c*_H2Oini_ of 5.3 wt%. The glass porosity of CD95 with *c*_H2Oini_ of 3.3 wt% H_2_O and a *P*_final_ of 20 MPa is below the minimum possible value (*Φ*_RES_). Possibly, in this sample equilibrium porosity was not achieved prior to quench. An H_2_O supersaturation prior to quench would counteract resorption until the solubility curve exceeds the residual *c*_H2O_ of the melt. Vesicle shrinkage would then start from a melt with porosity < *Φ*_equ_, but solely by EOS-shrinkage. However, according to the calculations above, diffusion must have been fast enough for equilibrium degassing and viscous retardation of vesicle growth is not expected due to a low viscosity of 10^3^–10^4^ Pa s (Thomas et al. 1994). However, according to Toramaru (1998) the effective viscosity of a vesicle-rich silicate melt is larger for pure melt with identical *c*_H2O_ if the fluid phase is stored in a large *VND* of small isolated vesicles. The two experiments with 3.3 wt% *c*_H2Oini_ are those with the highest log*VND* of ~ 6. This high *VND* might have caused a high bulk viscosity, limiting equilibrium growth of the vesicles. Furthermore, the sample decompressed to 20 MPa might have an unpredictably low glass porosity, because it was quenched close to the critical point of H_2_O, where slight changes in *T* and *P* have a large effect on fluid density and calculations close to this point might be prone to large errors. Therefore, these two experiments might not be suitable for the shrinkage calculations presented here, where degassing close to equilibrium prior to quench is a pre-requisite. Thus, the experiments with *c*_H2Oini_ of 3.3 wt% were not considered for the following calculations.

### Determination of *T*_f_ where vesicle shrinkage effectively stops

We conclude, that EOS and resorption driven vesicle shrinkage effectively stops at temperatures *T*_f_ > *T*_g_res_. This is based on the observation that *Φ*_glass_ of the investigated samples with 5.3 and 4.3 wt% *c*_H2Oini_ are higher than *Φ*_RES_ (Fig. 2). Different methods can be applied to determine *T*_f_:

#### Determination of *T*_f_ via glass porosity

**Fig. 4:**
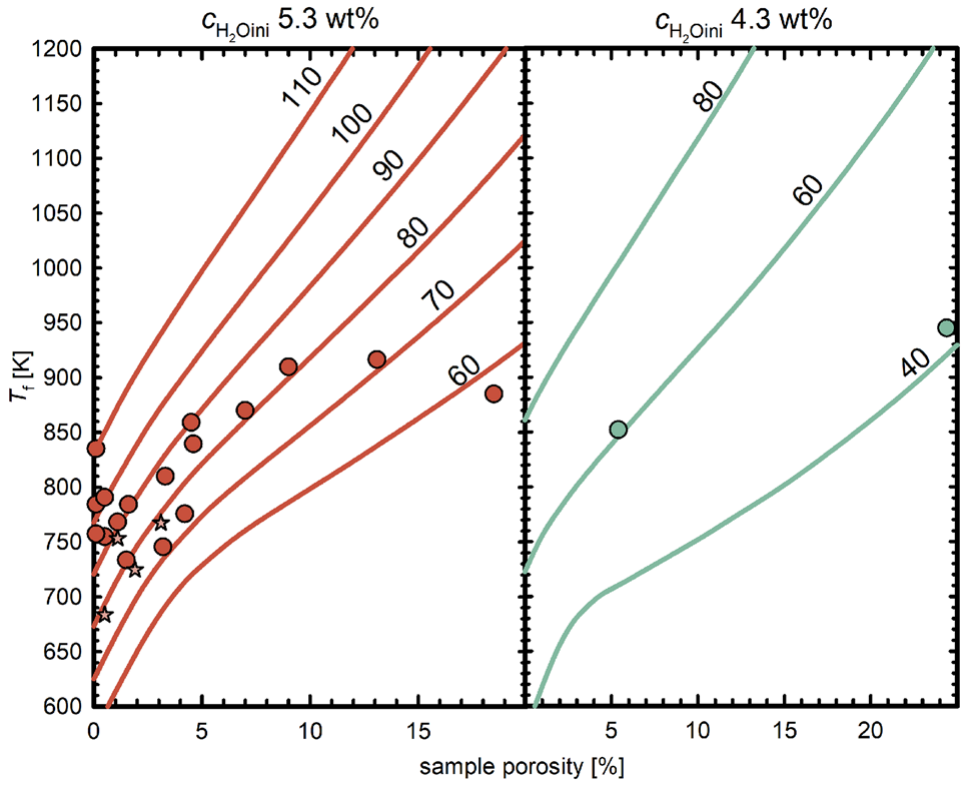
Determination of *T*_f_ from glass porosity. Temperature dependent equilibrium porosities are exemplarily calculated for 5.3 and 4.3 wt% *c*_H2Oini_ at the different *P*_final_ (given in MPa next to each line) at which the samples were quenched. *T*_f_ was calculated based on the glass porosity, *P*_final_ and the individual *c*_H2O_ of each sample. The data points show the *T*_f_ that is derived from glass porosity of each sample. They do not all plot exactly on the curves calculated for the respective *P*_final._ due to small deviation of *c*_H2Oini_ of the experiments from the H_2_O concentration that the curves were calculated for. Experiments with *c*_H2Oini_ of 3.3 wt% were not considered here because they may not have achieved equilibrium H_2_O content prior to quench, which however is a pre-requisite for the *T*_f_ calculation (for details see text)

For the determination of *T*_f_ from *Φ*_glass_, we assume that the observed glass porosity represents the equilibrium porosity of the supercooled melt at *T*_f_, where vesicle shrinkage effectively stops. Equation 7 can be used to calculate the *T* dependent *Φ*_equ,_ and therefore, we find the temperature *T*_f_ (and the resulting *ρ*_melt_, *V*_mH2O_ and H_2_O solubility) at known *P*_final_ and *c*_H2Oini_, where *Φ*_glass_ matches the calculated porosity (Fig. 4). The *T*_f_ for samples with 5.3 and 4.3 wt% *c*_H2Oini_ and a *q* of 44 K·s^−1^ range between 733 and 945 K, while *T*_f_ is lower at 16 K·s^−1^ with 683 and 767 K (Table 2).

#### Determination of T_f_ via the H_2_O liquid to vesicle volume ratio—application of XCT analysis

An independent method for determining the extent of vesicle shrinkage is provided by the ratio of liquid H_2_O volume (*V*_H2Ol_) in quenched vesicles to total vesicle volume (*V*_ves_) using XCT analysis (McIntosh et al. 2015).

During decompression, the exsolved H_2_O fluid is supercritical because *P*_final_ and *T* exceed the critical point of H_2_O. During cooling, vesicles shrink until *T*_f_ is reached. Below *T*_f_ the vesicle volume remains constant during further isochoric cooling and consequently, the pressure inside the vesicles starts to drop. The supercritical fluid follows a path of constant density until the liquid–vapor phase boundary is reached, after which the liquid–vapor ratio changes with the *P* in the vesicles following the water-steam equilibrium curve until ambient *T* is reached. H_2_O present as vapor and liquid at room temperature results in an internal vesicle *P* of 0.035 bar. The volume of liquid water (water vapor can be neglected in this case due to its low density) gives an approximation of the amount of H_2_O fluid trapped at *T*_f_. As the amount of liquid water observed at ambient *T* would completely fill the vesicle volume at *P*_final_ as a single phase fluid, we can calculate *T*_f_ from the equation of state of H_2_O. This method does not require knowledge of EOS- and H_2_O resorption-contribution to shrinkage.

**Fig. 5:**
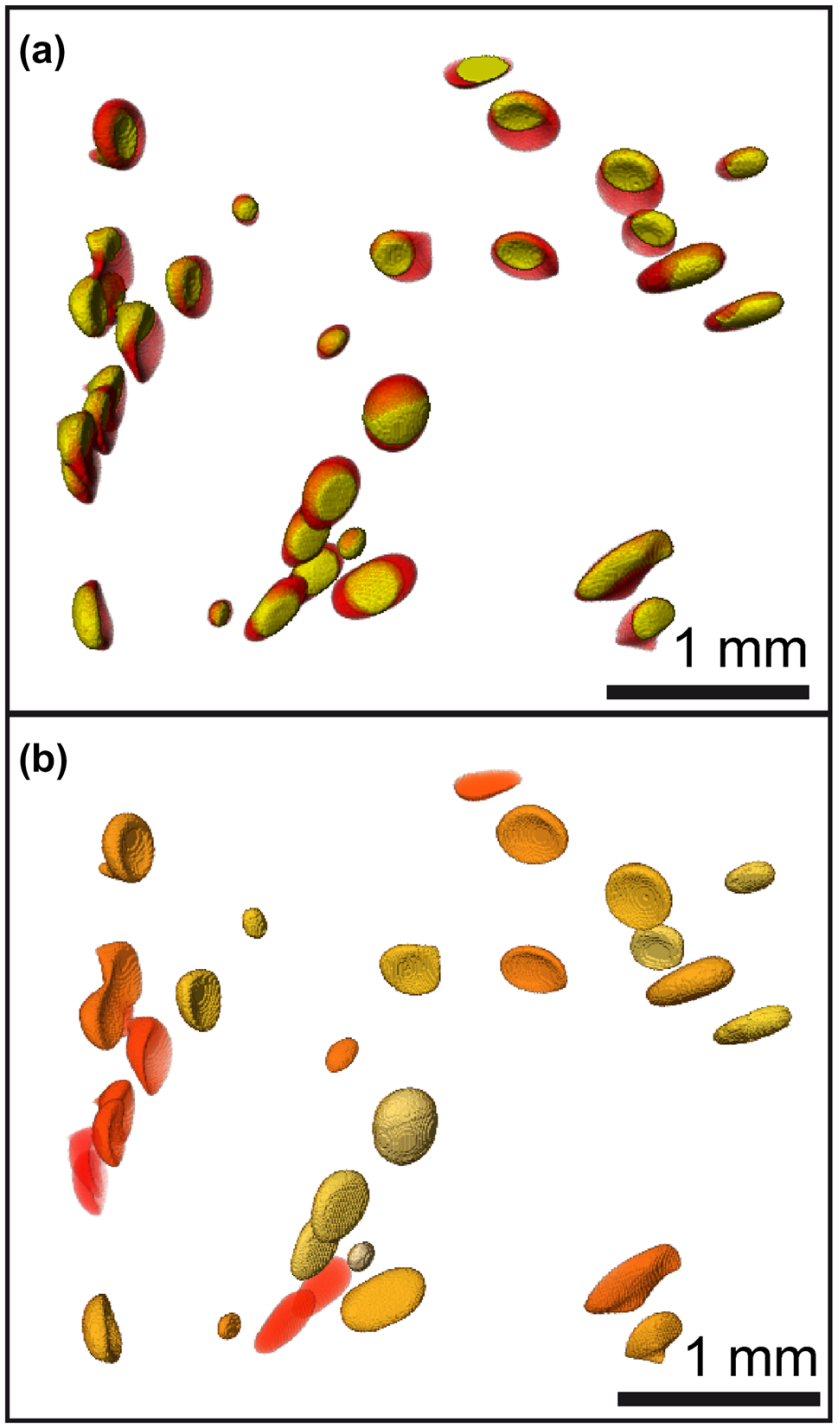
**a** XCT-images of large vesicles in the central vesiculated volume of CD74 that are filled with vapor (yellow) and liquid H_2_O (red) at ambient conditions. Volume of liquid H_2_O to vesicle volume ratios were used to calculate *T*_f_ where vesicle shrinkage stops. Due to capillary forces, the liquid water is not gravitationally located at the bottom of each vesicle. **b** same vesicles color coded for *T*_f_, with light yellow for high *T*_f_ towards orange for low *T*_f_. Edge length of the XCT images are approximately 3.6 mm

**Fig. 6:**
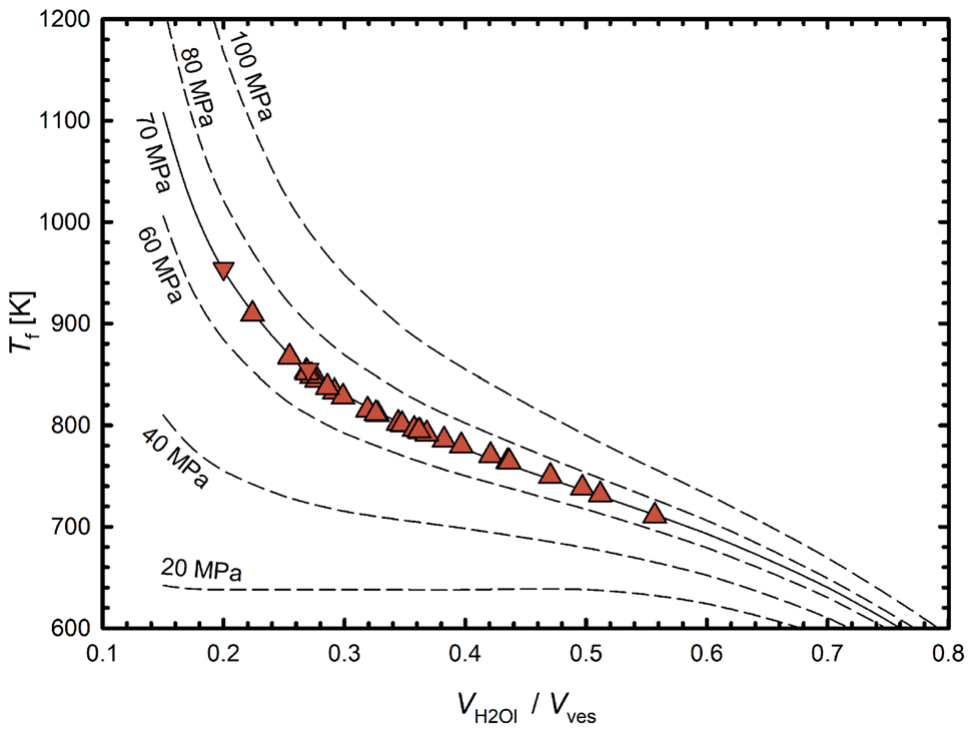
*T*_f_’s determined from individual large vesicles in CD74 using the ratio of the volume of liquid H_2_O in the vesicle to the total vesicle volume based on XCT data (Fig. 5) handled by automated analysis (triangles up) and manual analysis (triangles down). The determined volume ratios at *P*final of 70 MPa define a *T*_f_ range between 710 and 954 K for CD74. The additional dashed curves are exemplarily shown for different *P*_final_

**Fig. 7:**
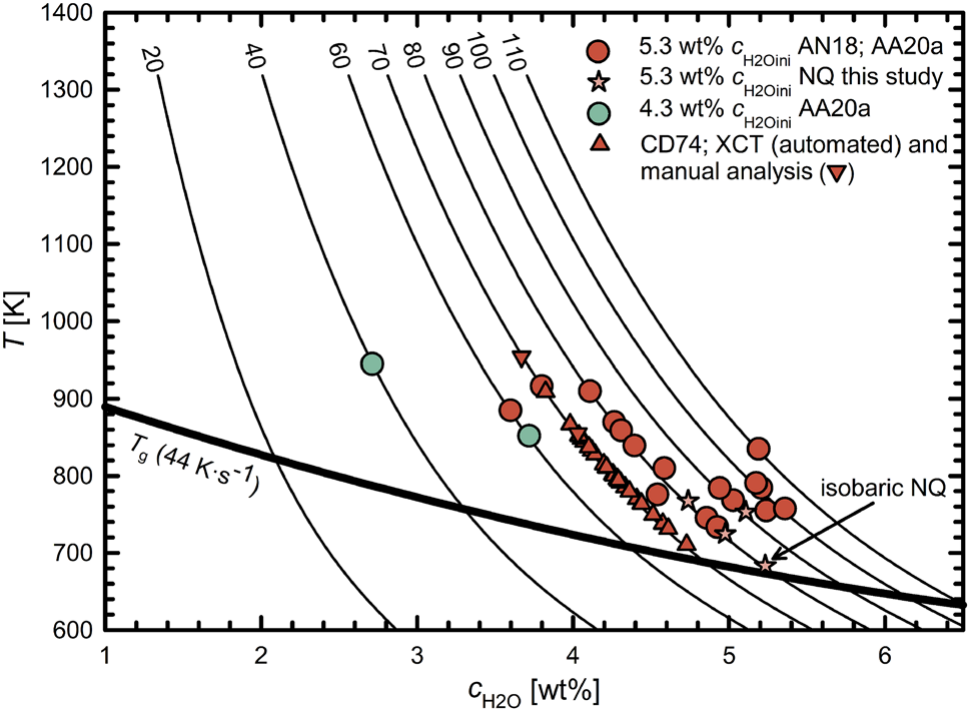
Summarized *T*_f_’s of samples calculated from both *Φ*_glass_ (Fig. 4) and XCT data (Fig. 6) in the same plot as Fig. 3. Each solubility curve (black lines) is labeled with the corresponding *P* in MPa. *T*_f_’s are up to 200 K higher than *T*_g_ which means that vesicle shrinkage stops significantly before *T*_g_ is reached. Isobaric NQ (16 K·s^−1^) reveals the lowest *T*_f_ because more time was available for vesicle shrinkage driven by H_2_O resorption and viscous flow of the melt compared to MQ (44 K·s^−1^). The XCT data based *T*_f_’s are qualitatively consistent with *T*_f_’s calculated from glass porosity. References: AN18: Allabar and Nowak (2018); AA20a: Allabar et al. (2020)

XCT-imaging reveals, that larger deformed vesicles are distributed within the central finely vesiculated volume of the MQ sample CD74 (Fig. 5a). The large vesicles may be ascended fringe vesicles or a product of coalescence (Allabar et al. 2020). The smaller dispersed spherical vesicles are close to the limit of XCT resolution and were therefore not included in the *V*_H2Ol_ analysis. Analysis of vesicles in CD74 yield *V*_H2Ol_ to *V*_ves_ ratios between 0.56 and 0.20, corresponding to *T*_f_ between 710 and 954 K, respectively (Fig. 6) with a random spatial distribution of *T*_f_ within the sample (Fig. 5b). The *T*_f_ estimates from XCT analysis are within the range of *T*_f_ derived from the glass porosity calculations (Fig. 7). The mean value of 810 K can be assumed, if cooling was homogeneous throughout the sample implying that a single temperature *T*_f_ is valid for the vesiculated part of the sample. However, we propose that vesicle shrinkage within an experimental sample may be a complex process including competition of vesicles in close vicinity (including the small, finely dispersed vesicle population) and the ability of a vesicle to achieve efficient volume reduction by deformation that will depend on its surrounding. These processes may lead to differences in *T*_f_ of individual vesicles.

Together, the methods of estimating *T*_f_ (Fig. 7) show that for samples with 5.3 and 4.3 wt% *c*_H2Oini_, vesicle shrinkage stops up to 200 K above *T*_g_, and that the relationship between *T*_f_ and *P*_final_ is similar to the relationship between *T*_g_ and *P*_final_. We assume that *T*_f_ derived from *Φ*_glass_ reveals a mean *T*_f_ for the whole central sample volume comparable to the mean *T*_f_ from XCT analysis. Using *T*_f_ and the *P*_final_ for each sample, the theoretical residual *c*_H2O_ in the supercooled melt at *T*_f_ (= *c*_H2Ores_Tf_) can be calculated (Fig. 7), and used to define the viscosity at which vesicle shrinkage stops. The viscosity *η*__Tf_ ranges from 10^6^ to 10^8^ Pa·s at 44 K·s^−1^ and appears to be independent of *P*_final_. For cooling with 16 K·s^−1^, we find a *η*__Tf_ of 10^8^ and 10^9^ Pa·s due to lower *T*_f_ as a result of slower cooling.

At lower viscosities (above *T*_f_) it is likely that the vesicles instantaneously shrink to their equilibrium size. This instantaneous growth and shrinkage has been shown in in-situ experiments (Masotta and Keppler 2014) demonstrating pressure cycling induced vesicle volume changes in a melt with a viscosity of 8.5·10^5^ Pa·s.

### Uncertainties of vesicle shrinkage calculations

The H_2_O-solubility equation (Eq. 8) used here is based on experimentally determined H_2_O solubility in phonolitic melt over *P* and *T* ranges between 20–395 MPa and 1123–1473 K (Shea 2017, Eq. 8 therein). Since there is no experimental access to low *T* and *P* H_2_O-solubility data in phonolitic melt between liquidus *T* and *T*_g_ due to crystallization, the applicability of this solubility model is uncertain, but remains up to now the only option to estimate H_2_O resorption. While it is assumed that towards lower *T*, phonolitic melts would exhibit a strong increase in H_2_O solubility as seen in haplogranitic melt (Shea 2017; Liu et al. 2005), this remains unconstrained.

An alternative H_2_O-solubility model, which is based on the assumption of thermodynamic equilibrium (Ottonello et al. 2018), allows further tests. However, it predicts a smaller increase of solubility with decreasing *T*, which contrasts with the high *c*_H2OIR_ measured in the glass between the vesicles (e.g., *c*_H2Ores_ of ~ 4.3 wt% calculated with the model for *P*_final_ = 80 MPa at *T*_g_res_, compared to ~ 5.3 wt% derived from Eq. 8 and ~ 5 wt% measured in the glasses). H_2_O resorption in samples of this study occurs in a *T* ranging between *T*_d_ and a temperature below the liquidus in the metastable state. In the H_2_O-solubility model of Liu et al. (2005) for rhyolitic melts, experimental data determined below the liquidus are included, which cannot be described by thermodynamic equilibrium. Since the H_2_O-solubility (Eq. 8) is based on these data, it is suggested that this dependence is more suitable for quantifying resorption processes of supercooled melt apart from equilibrium.

A further uncertainty in our calculations is that pure H_2_O is assumed to be in the vesicles rather than an H_2_O fluid with dissolved silicate components, which may influence the EOS parameters. However, at such low *P*, the solubility of melt components in H_2_O fluid is low (e.g., Stalder et al. 2000). Therefore, this will have only a minor influence on our calculations.

### H_2_O concentrations measured by FTIR and Raman micro-spectroscopy

**Fig. 8:**
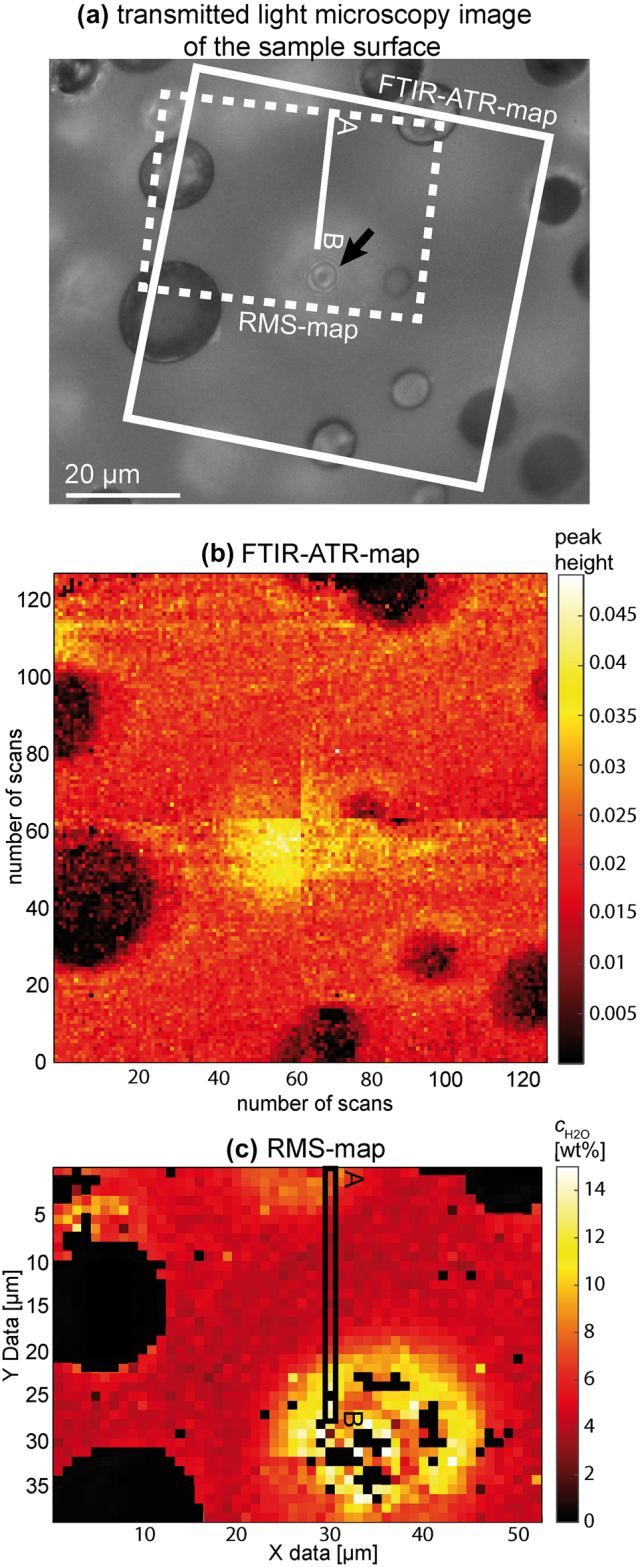
**a** Transmitted light microscopy image of the mapped area of CD73 focused at the sample surface. The arrow is pointing at the central intact vesicle that is located ~ 1 µm below the surface, as indicated by the light diffraction rings. The frames indicate the areas of FTIR-ATR and Raman micro-spectroscopy (RMS)-mapping. **b** FTIR-ATR map based on 128 × 128 MIR spectra representing a sample area of 70 × 70 µm stitched by 4 frames (64 × 64 spectra each) showing a slight lateral offset. Spectra of each 64 × 64 frame were monitored simultaneously with a focal plane array MIR detector. Quantitative *c*_H2O_ determination was not calibrated due to a small tilt of the Ge-ATR crystal relative to the sample surface. Therefore, the pixel are color coded for the peak height of the 3450 cm^−1^ fundamental OH stretching band, representing total dissolved H_2_O near the surface of the intersected sample. **c** RMS-map: color coded *c*_H2O_ concentrations derived from Rama spectra. RMS measurements that are affected by epoxy or carbon relicts (mostly within the intersected vesicles from preparation and previous carbon coating for SEM imaging), are colored black

The results of the FTIR-ATR mapping and RMS mapping on sample CD73 are shown in Fig. 8. In both the FTIR-ATR-map and the RMS-map an increase in *c*_H2Oglass_ towards vesicles is detected, testifying H_2_O resorption during cooling. With both methods, this can be clearly seen in the case of the vesicle just below the glass surface (marked with an arrow in Fig. 8a), with a resorption halo intersecting the sample surface. However, the results of the two methods differ with respect to the area of increased *c*_H2Oglass_ above this vesicle that appears to be larger in the RMS-map. Additionally, the resorption halos with increased *c*_H2Oglass_ around intersected vesicles are clearly visible only in the FTIR-ATR-map. We attribute this to the different beam penetration depth of the two methods. In case of the FTIR-ATR measurements, the effective penetration depth is ~ 0.25 µm (calculation after Compton and Compton (1993) given in the online resource “RMS and FTIR supplementary information”). Penetration depth of the RMS measurement must be higher to explain the RMS data. To get an estimate on RMS-penetration depth, we used a VAD79 thin section that was polished to ~ 20 µm thickness and measured it on top of a Si single-crystal standard that is usually used for the performance check of the spectrometer. With the same measurement conditions as for the mapping of the vesiculated samples, we find a distinct Si-Raman signal although the laser beam was focused at the glass sample surface. Thus, the penetration depth of the laser is > 20 µm. However, the effective penetration depth that quantitatively determines the *c*_H2O_ result is unknown. The large laser penetration depth is consistent with the study of Everall (2000), who finds that depth resolution of Raman spectroscopy is limited and usually worse than expected. Depth resolution gets worse by focusing into the sample volume, such that a 5 µm focus depth results in an illuminated depth of ~ 18 µm at a refractive index of 1.5 (Everall 2000). For glasses with homogeneously dissolved H_2_O, it is usually suggested to use an optimal focus depth where HW and LW bands are at a maximum intensity (~ 5–10 µm focus depth depending on glass composition; Schiavi et al. 2018; Di Genova et al. 2017) because this reduces the error in *c*_H2O_ by focus inaccuracies. However, this approach should not be used for samples inhomogeneous in *c*_H2O_ for which a high spatial resolution *and* depth resolution is desired.

**Fig. 9:**
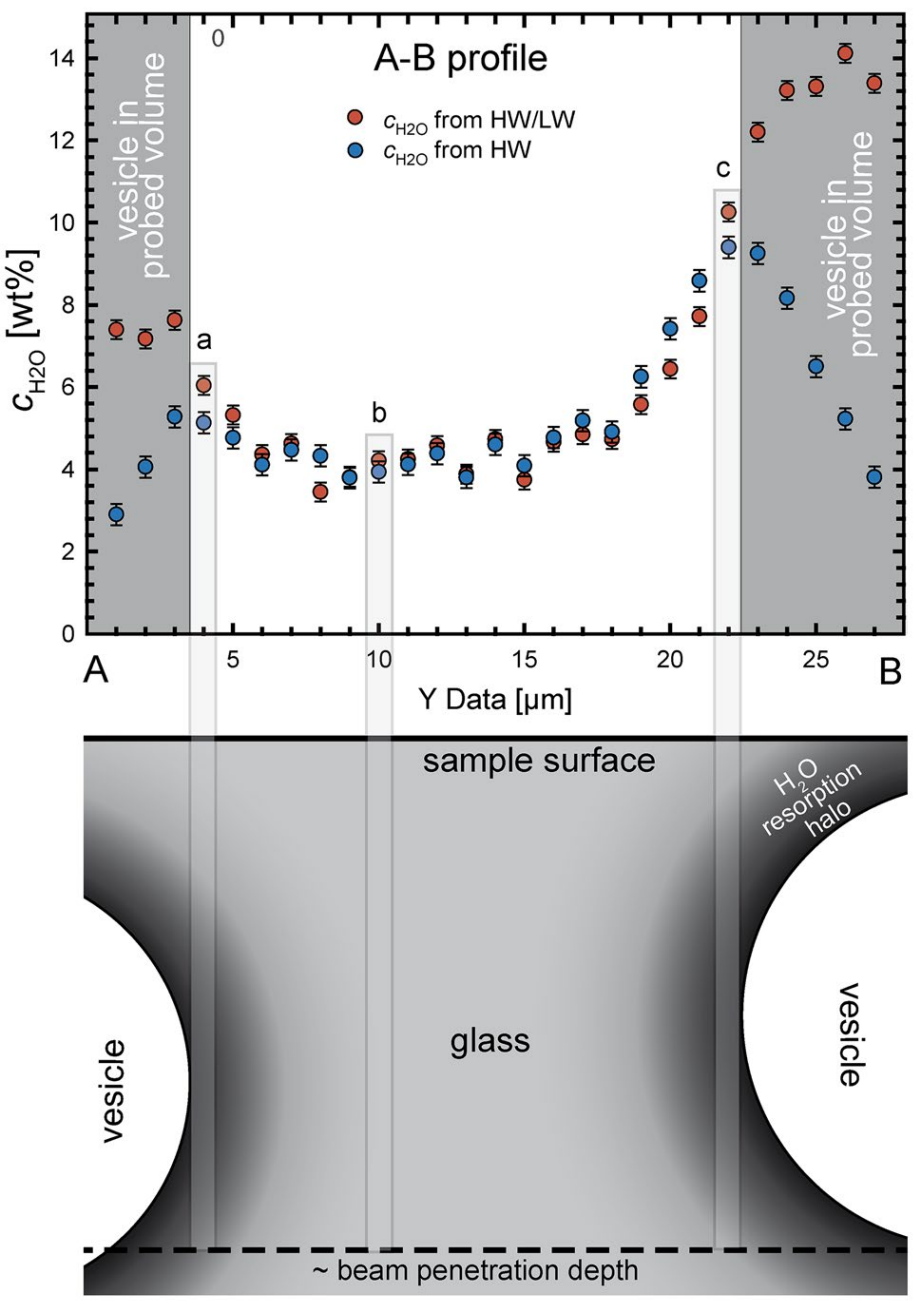
Raman measured *c*_H2O_—distance profile from position A to B as indicated in Fig. 8a, c. *c*_H2O_ from both HW and HW/LW calibration, is shown. Errors in *c*_H2O_ relate to the errors of calibration using hydrous VAD79 samples with different *c*_H2O_ (for details see online resource “RMS and FTIR supplementary information”). Below is a schematic illustration of the AB intersection of the sample below the surface. The vesicle at profile end A is positioned slightly deeper than the vesicle at the end B, which is ~ 1 µm below the surface. In the schematic intersection, the vesicle is drawn slightly deeper because the profile approaches the vesicle slightly lateral. The penetration depth of the laser beam during RMS measurements was determined to be at least 20 µm as indicated by the dashed line. Thus, the measurements with a vesicle in the probed beam yield different *c*_H2O_ values, dependent on HW and HW/LW calibration (data in grey areas; for details see text). All other measurements are unaffected by the vesicles. The highlighted bars indicate the illuminated volume yielding the resulting *c*_H2O_ to which they are connected in the plot above. The *c*_H2O_ at position a is lower than at position c because the high *c*_H2O_ of the H_2_O resorption halo (shaded in black to dark grey) around the vesicle A is more diluted by H_2_O poorer glass towards the sample surface compared to vesicle B

The large penetration depth using RMS of at least 20 µm has to be considered for the interpretation of *c*_H2Oglass_ between vesicles. In detail, a *c*_H2O_ profile extracted from the RMS-map between two vesicles below the surface is shown by the A-B profile (Figs. 8a, c, 9). In transmitted light, a diameter of ~ 20 µm of the vesicles below the surface of the A-B profile was determined. The vesicle at the profile end B is ~ 1 µm below the surface, which is also indicated by the diffraction rings on the transmitted light microscopy image of the sample surface (Fig. 8a). The other vesicle at B is slightly deeper (Fig. 9). In the A-B profile (Fig. 9) the *c*_H2O_ determined from the HW calibration (3450 cm^−1^ band only, i.e., total H_2_O) decreases when approaching vesicles that are several microns below the surface, while the *c*_H2O_ from HW/LW calibration further increases. The latter is related to the HW/LW calibration that reflects H_2_O concentration per glass volume and is thus independent from the measured glass volume. A lower *c*_H2O_ near the vesicle at the profile end A is measured because the vesicle is located deeper below the sample surface. Compared to the vesicle at profile end B, which is closer to the surface (Fig. 9), a larger glass volume with less H_2_O is measured at profile end A. This dilutes the Raman signal of resorption halos near the vesicle (Fig. 9). The same dilution problem occurs when measuring in the vicinity of intersected vesicles. Because of the large penetration depth of Raman spectroscopy measurements, the increased *c*_H2Oglass_ around the vesicle, which extends only a few micrometers away from the vesicle, is diluted by H_2_O poorer glass underneath (Fig. A3 in online resource “RMS and FTIR supplementary information”). Thus, resorption halos of intersected vesicles are clearly visible only in the FTIR-ATR-map.

Despite the high penetration depth, we can use the H_2_O contents from the RMS-map, which are not influenced by a vesicle in the measured volume. Theoretically, the HW/LW values with vesicles in the probed volume represent the H_2_O content in the glass above the vesicle. However, we do not know whether the vesicle wall has a reflectance effect on the laser beam, which could distort the signal. Because of this uncertainty, we only consider measurements without vesicles in the probed volume. This is the case for measurements for which the HW and HW/LW calibration provide identical results within error (Fig. 9). We exclude all measurements from further interpretation, where the deviation of the two values is greater than the relative change to the nearest value (Fig. 9, data with grey background). In the sample volume between the vesicles, a relatively homogeneous *c*_H2O_ of ~ 4.3 wt% is measured, which is significantly higher than calculated equilibrium *c*_H2O_ of 2.6 wt% at *P*_final_. McIntosh et al. (2014) found ~ 20 µm diffusion profiles (with 12 µm half fall distance) in a phonolitic sample after rapid cooling within 3–10 s at 100 MPa. Therefore, it can be assumed that sample CD73 with diffusion lengths of ~ 10 µm and cooling within ~ 20 s has adjusted to the measured *c*_H2Oglass_ of 4.3 wt% between the vesicles by resorption, although the melt had a low equilibrium *c*_H2O_ of ~ 2.6 wt% at *P*_final_ prior to cooling. We attribute the steep increase of *c*_H2O_ towards the vesicles to resorption at a late stage, where solubility strongly increases and concurrently, diffusivity decreases during cooling, becoming too slow for equilibrating the entire supercooled melt volume between the vesicles. Thus, the shape of such diffusion profiles results from the interplay of the timescale of cooling with the H_2_O solubility increase and diffusivity decrease.

All *c*_H2O_ values measured between the vesicles of CD73 are above the calculated *c*_H2Ores_Tf_. This is also the case for the NIR-FTIR transmission measurements through the glass without vesicles in the beam (Table 2). These measurements resemble a mean *c*_H2O_ between vesicles, but with unknown contribution of steep H_2_O gradients towards vesicles. Nevertheless, the general observation of higher *c*_H2O_ in the glass compared to calculated values may indicate, that further H_2_O diffusion occurs below *T*_f_ without further vesicle shrinkage (McIntosh et al. 2014). Assuming isochoric behavior of vesicles below *T*_f_, H_2_O diffusion from fluid to melt or glass below *T*_f_ changes the proportions of H_2_O liquid and vapor observed at ambient *T* and may affect the *T*_f_ determination using XCT data. To test this, the liquid H_2_O proportion in small vesicles of the pervasive clouds need to be analyzed in the future, where resorption is expected to be much more effective due to short diffusion distance than in the large vesicles that were used here. Alternatively, the discrepancies between *c*_H2Ores_Tf_ and *c*_H2O_ measured in the glasses can also originate from an underestimation of H_2_O solubility towards low *T* by Eq. 8. Additionally to the discussion of experiment CD95 above, this could be an alternative reason for the mismatch between calculated and observed porosity.

### Impact of experimental technique on vesicle shrinkage

**Fig. 10:**
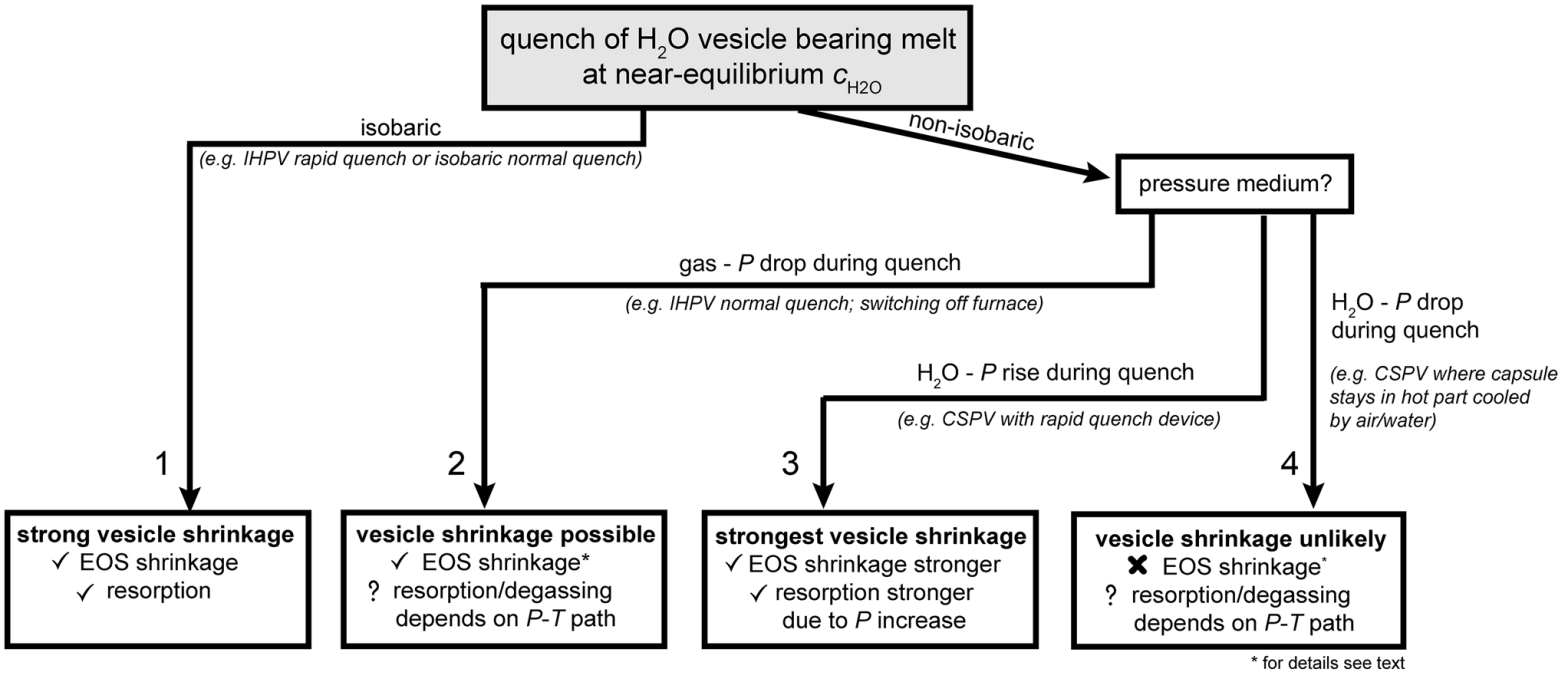
Flowchart for illustrating the impact of different experimental quench techniques on H_2_O-vesicle shrinkage in melts that reached near equilibrium *c*_H2O_ prior to quench. For a constant cooling rate, vesicle shrinkage and the single contributions (EOS- and resorption-shrinkage) will vary according to the shown quench conditions

Quantification of vesicle shrinkage in post-mortem analyzed H_2_O-vesicle bearing samples requires knowledge of the experimental apparatus and the quench technique used (Fig. 10). When samples are quenched isobarically, EOS-shrinkage and resorption will be driven by *T* drop only (case 1 in Fig. 10). For a non-isobaric quench, shrinkage will depend on the pressure medium that surrounds the sample and on the magnitude of pressure drop. When gas is used as pressure medium, *P* drops during cooling (CD49, this study). However, the *P* drop within the autoclave is less than that expected from a simple EOS calculation of the gas because only part of the gas volume of the IHPV is heated prior to quench. Nevertheless, *V*_mH2O_ decreases stronger during cooling than the *V*_m__gas_ of the pressure medium. The pressure inside the vesicle will drop relative to the pressure medium and EOS shrinkage is facilitated (case 2). Test experiments in cold seal pressure vessels (CSPV), using H_2_O as pressure medium, revealed that *P* may rise by up to 10 MPa during cooling when a rapid quench device is used because the hot capsule and parts of the guide rod heats the water in the cold part of the vessel by dropping in it. In this case, the strongest vesicle shrinkage is possible because the *P* increase supports resorption by solubility increase and compression of H_2_O according to the EOS (case 3). If the sample remains in the hot section and the vessel is cooled by compressed air or water externally (e.g., Larsen and Gardner 2004), both the pressure inside the autoclave and the vesicles will drop during cooling according to EOS of H_2_O, provided the *P* is not actively held constant. Because there is no *P* difference between the vesicles and the pressure medium, there will be no driving force for EOS-shrinkage (case 4). However, during cooling accompanied with a *P* drop, the retrograde *T* dependence of H_2_O solubility will compete with the *P*-induced solubility decrease. Shrinkage in such an experiment is unlikely or will be of minor extent. However, the prerequisite for shrinkage to occur at all is that some time for melt relaxation and diffusion is given. Theoretically, a sample has to cool infinitely fast to prevent vesicle shrinkage. The experiments summarized in this study belong to case 1 (MQ and NQ isobaric) and case 2 (CD49).

### Review of experimentally derived porosities in phonolitic melt

Numerous decompression experiments have been performed using hydrous phonolitic melts (Larsen and Gardner 2004; Iacono-Marziano et al. 2007; Larsen 2008; Mongrain et al. 2008; Shea et al. 2010; Gardner 2012; Marxer et al. 2015; Preuss et al. 2016; Allabar and Nowak 2018; Allabar et al. 2020). When these data are compiled with the data of this study (online resource “Literature review”) we can evaluate the effect of vesicle shrinkage during cooling also in these experiments, which are quenched with more commonly used rapid quench protocols (> 100 K s^−1^). We include only such data where H_2_O was the only volatile, degassing occurred in a closed system and glass porosity and *VND* were reported for experiments quenched at *P*_final_ > 22.1 MPa (above the critical point of H_2_O).

**Fig. 11:**
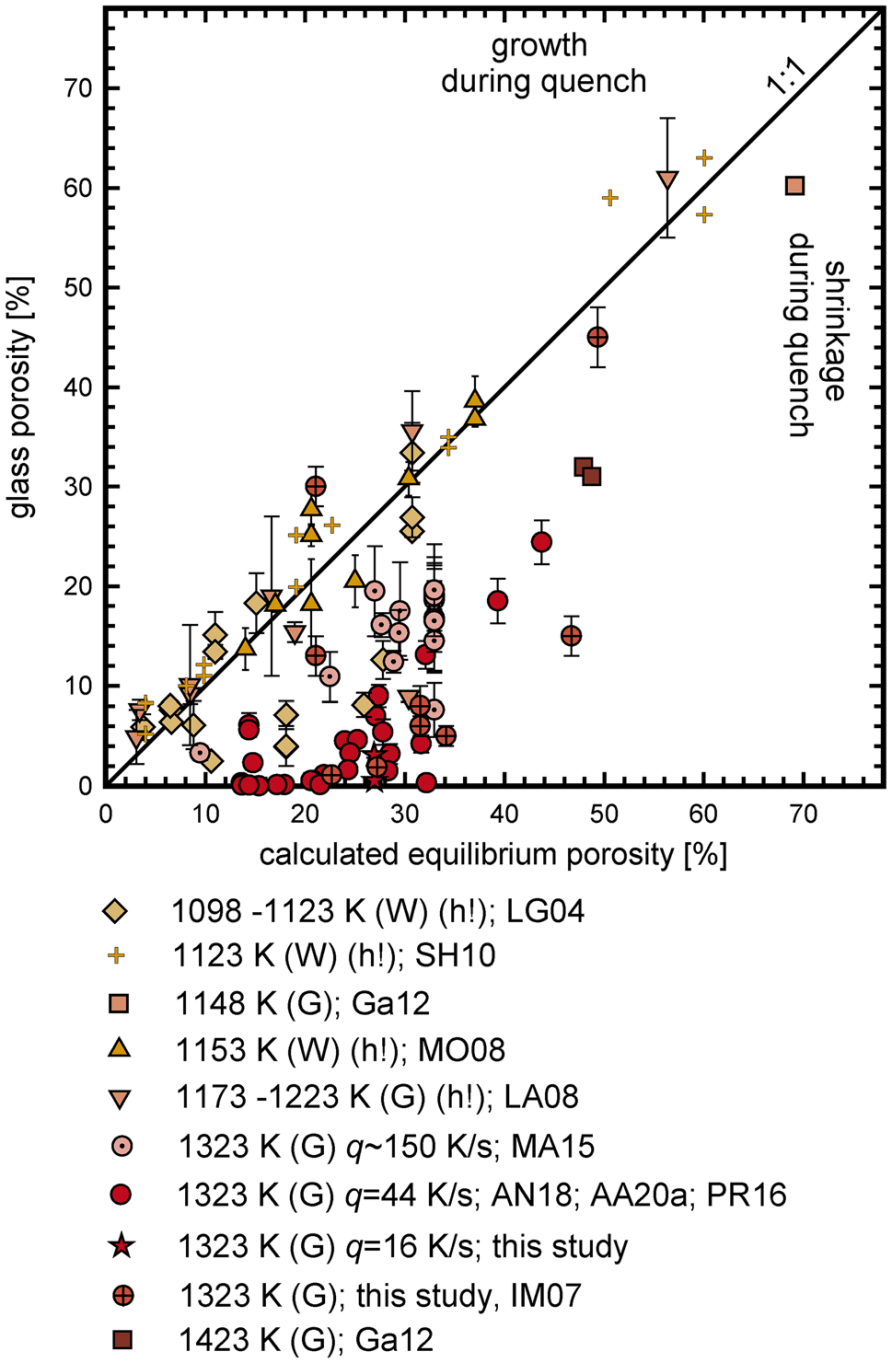
Observed glass porosities vs. calculated equilibrium porosities for all decompression experiments with hydrous phonolitic melt composition, in which near-equilibrium degassing was facilitated prior to quench. (G) gas as pressure medium, (W) water as pressure medium. When *q* and error bars are not given, they are unknown. (h!) hydration vesicles might have been present prior to decompression due to the use of powder as starting material and H_2_O supersaturated conditions prior to decompression. The experiments with melts containing hydration vesicles are the only ones plotting close to the 1:1 line, suggesting that no vesicle shrinkage occurred. The presence of a pre-existing fluid phase, however, can shift the glass porosities of the quenched samples to higher values towards or above the 1:1 line, although shrinkage occurred. Additionally, other reasons might also influence the glass porosity of these samples (see text). All data of other samples plot below the 1:1 line indicating vesicle shrinkage during cooling. A faster cooling rate (150 K·s^−1^) leads to slightly less shrinkage (MA15) compared to slower cooling (44–16 K·s^−1^; this study) References: LG04: Larsen and Gardner (2004); SH10: Shea et al. (2010); GA12: Gardner (2012); MO08: Mongrain et al. (2008); LA08: Larsen (2008); MA15: Marxer et al. (2015); AN18: Allabar and Nowak (2018); AA20a: Allabar et al. (2020); PR16: Preuss et al. (2016); IM07: Iacono-Marziano et al. (2007). Compilation of data can be found in the online resource “Literature Review”

For the compiled experimental data, we calculated whether near equilibrium H_2_O content can be adjusted prior to quench (see calculations described above). Therefore, when available, the solubility data of each study were used for H_2_O solubility at given run conditions. Otherwise, the general H_2_O-solubility dependence for phonolitic melt (Eq. 8) was used and H_2_O diffusivity was calculated with Eq. 3 (Fanara et al. 2013). For all experiments, in which near-equilibrium porosity was adjusted prior to quench, the equilibrium porosity was calculated. The *Φ*_glass_ vs. *Φ*_equ_ is shown in Fig. 11 . In about half of the decompression experiments, vesicle shrinkage has likely occurred during cooling (*Φ*_glass_ < *Φ*_equ_), despite faster quench rates (150–200 K s^−1^) than in our study. The occurrence of vesicle shrinkage even at these high cooling rates is consistent with the large resorption-induced H_2_O gradients towards vesicles rims found by McIntosh et al. (2014) in phonolitic glasses after cooling within 3–10 s from *T*_d_ to ambient *T*. The varying degrees of vesicle shrinkage presented in in Fig. 11 can be attributed to the following parameters:

#### Different experimental techniques

Experiments from studies using gas as pressure medium ((G) in Fig. 11) experienced a near isobaric quench (Case 1, Fig. 10), and showed large degrees of vesicle shrinkage, varying with quench rate (Fig. 11). For most studies using H_2_O as pressure medium ((W) in Fig. 11) the actual quench rates and *P* changes during cooling are not known. Thus, direct comparison is not possible. One study reports a slight *P* increase of 5 MPa during quench (Case 3, Mongrain et al. 2008). Therefore, stronger vesicle shrinkage is expected but not observed (Fig. 11). This is likely due to higher cooling rate (> 100 K/s) compared to the studies using gas as pressure medium and the possibility of pre-existing hydration vesicles (see below).

#### Run temperature

*Φ*_equ_ increases with *T*_d_ and consequently the difference between the experimental *T*_d_ and *T*_f_ increases, enhancing vesicle shrinkage. The compiled data suggest an increase in vesicle shrinkage with increasing *T*_d._ However, several mechanisms contribute to shrinkage, and run temperature is not solely responsible for the varying degree of vesicle shrinkage.

#### Hydration vesicles prior to decompression

For some experiments, glass powder was used as starting material. During hydration, H_2_O vesicles can remain in the hydrated melt at H_2_O supersaturated conditions prior to decompression, resulting in a melt with up to 12% porosity prior to decompression (Larsen 2008). These pre-existing fluid vesicles can cause artificially high glass porosities close to, or above the equilibrium line. Vesicle shrinkage might therefore still have occurred during cooling.

#### Crystallization

Some experiments (Mongrain et al. 2008; Larsen and Gardner 2004; Larsen 2008; Shea et al. 2010) were performed below the liquidus and crystals are reported or documented in the SEM images. Crystals increase the bulk viscosity (e.g., Costa 2005), which could decrease the degree of vesicle shrinkage due to the shift of *T*_f_ towards higher values.

## Conclusion

Significant vesicle shrinkage can occur during cooling of vesicle-bearing silicate melts that achieved near-equilibrium *c*_H2O_ during decompression. The degree of vesicle shrinkage (EOS- and H_2_O-resorption shrinkage) varies with experimental technique and quench style, with shrinkage enhanced by high residual *c*_H2O_ in the melt (i.e., *P*_final_), increasing *T*_d_, and slower quench rates.

The procedures presented here demonstrate how vesicle shrinkage can be determined, and how we can gain insight into the processes controlling shrinkage for experimentally vesiculated and quenched samples. While we can extract semi-quantitative information that constrains whether melt degassing is occurring in equilibrium or disequilibrium, we highlight the need for improved understanding of EOS- and resorption shrinkage through both in-situ decompression experiments and numerical modelling.

The present study was limited to phonolitic melts, but while the processes controlling shrinkage will be less pronounced in rhyolitic melts, they should still be important, and shrinkage will be especially significant for decompression experiments on melts with high H_2_O diffusivity and low melt viscosity. It is therefore critical to consider vesicle shrinkage before porosity and *c*_H2O_ data from post-mortem samples of decompression experiments are used for interpreting vesiculation of silicate melts. Otherwise, melt porosity is significantly underestimated leading to a false interpretation of the onset of coalescence, percolation or the distinction between equilibrium or dis-equilibrium degassing of a melt.

## Electronic supplementary material

Below is the link to the electronic supplementary material.

Supplementary file1 (XLSX 61 kb)

Supplementary file2 (PDF 1990 kb)
