# Supplementary material for: Vesicle shrinkage in hydrous phonolitic melt during cooling
Source: Contrib Mineral Petrol. 2020 Feb 12;175(3):21. doi: 10.1007/s00410-020-1658-3 (PMC7080310; doi:10.1007/s00410-020-1658-3)
Supplement: Supplementary file 2 — Supplementary file2 (PDF 1990 kb) [file 410_2020_1658_MOESM2_ESM.pdf]

RMS and FTIR supplementary information

"Vesicle shrinkage in hydrous phonolitic melt during cooling"

Contributions to Mineralogy and Petrology

A. Allabar A[1], K.J. Dobson, C.C. Bauer, M. Nowak

[1]anja.allabar@uni-tuebingen.de; ORCID iD 0000-0003-2137-8590

Department of Geosciences, University of Tübingen, Germany

Wilhelmstraße 56, 72074 Tübingen

DOI: 10.1007/s00410-020-1658-3

Calibration of Raman micro-spectroscopy (RMS) for quantitative H_2O determination

Calibration was performed using the hydrated VAD79 samples REF02-06 from Marxer et al. (2015) and an unpublished sample that was hydrated with 1.37 wt% H_2O. H_2O concentrations are known from FT-NIR measurements of Marxer et al. (2015). For each standard sample 4-7 RMS spectra were acquired, part of which were measured before and part of which was measured directly after the mapping, without changing the optical setting of the instrument. Measurement setting for the calibration were the same as for the mapping of CD73 presented in the paper: a 50x objective with a numerical aperture of 0.75 was used, and spectra were collected from 100-4000 cm^{-1} in high confocality setting with an acquisition time of 10 s. Spectra were corrected for frequency-dependent scattering intensity of the laser and the temperature and baseline subtracted using the Matlab©-script provided by Di Genova et al. (2017). After this treatment, the Matlab©-script calculates the band area of the low wavenumber (LW, 200-1250 cm^{-1}) alumino-silicate bands and the fundamental OH stretching band at high wavenumber (3450 cm^{-}1; HW) representing total dissolved H_2O. Baseline substraction is exemplarily shown in Fig. A1.

Table A1 Hydrated VAD79 reference samples (Marxer et al. 2015) used for c_{H2O} calibration of Raman spectroscopy data. Samples were hydrated in the IHPV at 1323 K and 200 MPa and were equilibrated for 96 h.

sample	c_{H2O} (FTIR) [wt%]	absolute error in c_{H2O} [wt%]
REF01	1.37	0.13
REF02	2.20	0.16
REF03	3.10	0.21
REF04	4.10	0.28
REF05	5.15	0.36
REF06	5.60	0.40

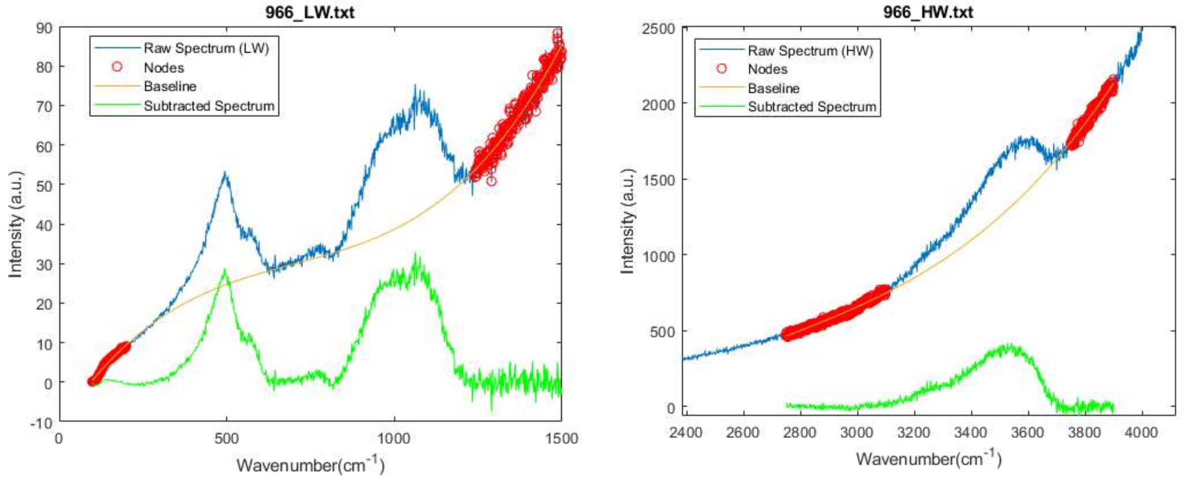

Fig. A1 Images generated by the Matlab©-Script provided by DiGenova et al. (2017). The images show raw Raman spectra (blue) and baseline substracted Raman spectra (green) of the LW band (left) and HW water band (right).

We used two types of calibration: (1) Calibration of the H_2O band (HW) area, which is also known as "external calibration" and (2) the ratio of the HW and the LW band areas (HW/LW), also known as "internal calibration" (Schiavi et al. 2018). However, we calibrated both, HW and HW/LW against the external standards. Weighted least-squares linear regressions (Fig. A2) with R^2 of 0.97 and 0.98, respectively, are obtained for the homogeneous hydrous VAD79 standard glasses (Table A1) and the given measurement settings. The linear dependency of HW/LW vs. c_{H2O} from FTIR measurements was used to process the mapped Raman data of sample CD73.

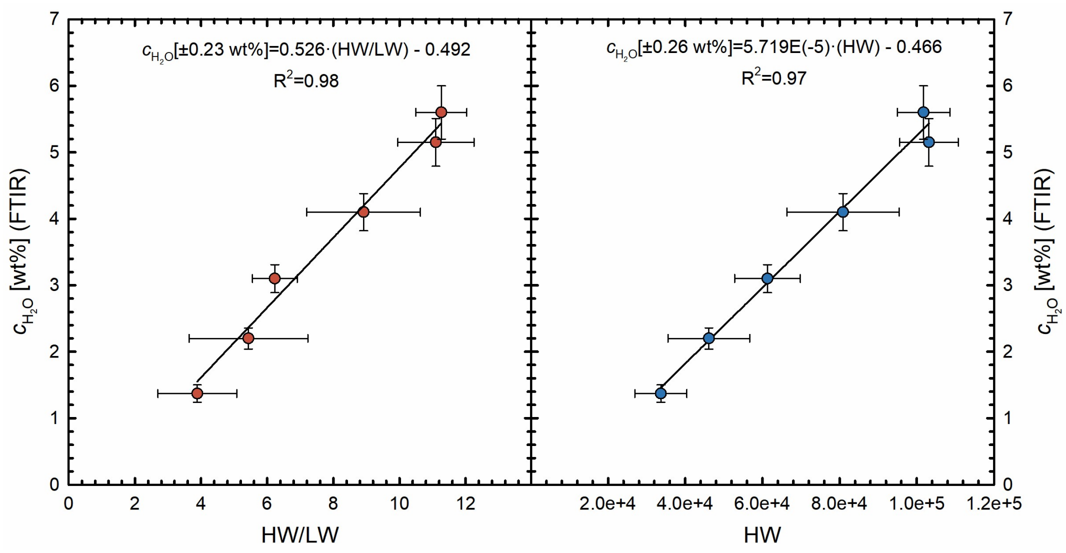

Fig. A2 Weighed least-squares linear regressions of HW/LW and HW band calibration to c_{H2O} from FTIR. Error bars in c_{H2O} are absolute errors from FT-NIR-H_2O determination. Error bars in HW/LW and HW band area indicate the standard deviation from multiple measurements.

Penetration depth during H_2O mapping of partly degassed phonolitic glasses

FTIR- ATR spectroscopy

The effective penetration depth (d_p) of the evanescent wave during ATR measurement with FTIR was calculated for the Bruker Hyperion3000 microscope equipped with a Ge-crystal ATR objective (20x) after Compton and Compton (1993):

$$d_p = \frac{\lambda}{2\pi n_p (sin^2\theta - n_{sp}^2)^{\frac{1}{2}}} \quad (1)$$

where λ is the wavelength of radiation in the Ge-crystal, θ ist the angle of incidence, n_p the refractive index of the ATR Ge-crystal (=4.0) and n_{sp} is the refractive index ratio of the glass sample and the ATR crystal. The angle of incidence in the optical setup used in this study is fixed at ~37°. Using the linear dependency of glass density as a function of H_2O concentration (c_{H2O}) for phonolitic glass (Eqn. 2; Iacono-Marziano et al. (2007) yields glass a density of ~2.4 $g \cdot cm^{-3}$ for 3.5-6 wt% H_2O.

$$\rho\left[\frac{g}{cm^3}\right] = 2.47 - 0.013 \cdot c_{H2O} \quad (2)$$

The corresponding refractive index determined from Tan and Arndt (2001) is ~1.5 at a light wavelength of 0.59 µm. The refractive index decreases only slightly with increasing wavelength (Tan and Arndt 2001). Thus, 1.5 is used to calculate penetration depth for the 3450 cm^{-1} water band (λ=2.899 µm), which yields a d_p of 0.25 µm.

Comparison of penetration depth using Raman and FTIR-ATR spectroscopy

The different penetration depths of RMS and FTIR-ATR spectroscopy explain the differences in relative H_2O concentration changes of the 2D maps that are obtained from both methods. In the RMS-map no increase in c_{H2O} around intersected vesicles is observed (Fig. A3), while such an increase in c_{H2O} is found with FTIR-ATR spectroscopy.

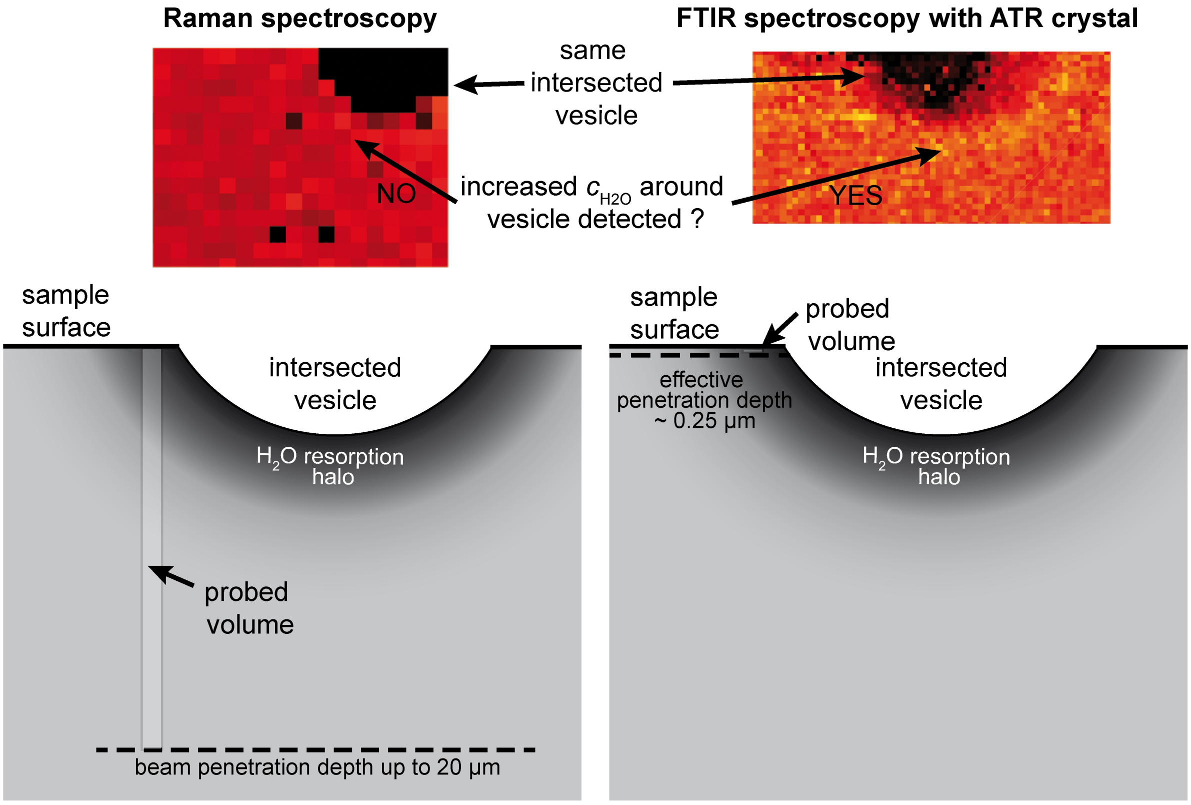

Fig. A3 Schematic comparison of penetration depth of confocal Raman spectroscopy measurements on sample CD73 with FTIR-ATR explaining why an increased c_{H2O} is detectable around intersected vesicles with FTIR-ATR-FPA mapping, but not with Raman spectroscopy.

The sample volume probed by Raman spectroscopy is ~20 µm deep and the high c_{H2O} in the resorption halo is diluted by the signal of the glass below with less H_2O. In contrast to that, using the FTIR-ATR method with the low penetration depth, the increase in c_{H2O} close to the vesicle can be detected (Fig. A3). Concluding, with both methods H_2O-gradients of vesiculated silicate glasses can be detected. Although it is not clear what the effect of vesicle walls within the laser-excited sample volume is, Raman spectroscopy reveals depth information that can be well quantified, at least for the vesicle free regions of the glass. FTIR-ATR spectroscopy is more suitable for surface-near high spatial resolution measurements. However, perfectly flat sample surfaces and tilt-free alignment with the ATR-crystal are required.

References

Compton SV, Compton DAC (1993) Optimization of data recorded by internal reflectance spectroscopy. In Coleman PB, Ed, Practical Sampling Techniques for Infrared Analysis, 55–92. CRC Press, Boca Raton, Florida

Di Genova D, Sicola S, Romano C, Vona A, Fanara S, Spina L (2017) Effect of iron and nanolites on Raman spectra of volcanic glasses: A reassessment of existing strategies to estimate the water content. Chem Geol 475:76-86

Iacono-Marziano G, Schmidt BC, Dolfi D (2007) Equilibrium and disequilibrium degassing of a phonolitic melt (Vesuvius AD 79 "white pumice") simulated by decompression experiments. J Volcanol Geotherm Res 161:151–164

Marxer H, Bellucci P, Nowak M (2015) Degassing of H_2O in a phonolitic melt: a closer look at decompression experiments. J Volcanol Geotherm Res 297:109–124

Schiavi F, Bolfan-Casanova N, Withers AC, Médard E, Laumonier M, Laporte D, Flaherty T, Gómez-Ulla, A (2018) Water quantification in silicate glasses by Raman spectroscopy: Correcting for the effects of confocality, density and ferric iron. Chem Geol 483:312-331

Tan CZ, Arndt J (2001) The refractive index of silica glass and its dependence on pressure, temperatre, and the wavelength of the incident light. In Nalwa HS, Ed, Silicon-Based Materials and Devices 2, Chapter 2, 50-91. Academic Press, New York
